# A Myc-driven self-reinforcing regulatory network maintains mouse embryonic stem cell identity

**DOI:** 10.1038/ncomms11903

**Published:** 2016-06-15

**Authors:** Luca Fagnocchi, Alessandro Cherubini, Hiroshi Hatsuda, Alessandra Fasciani, Stefania Mazzoleni, Vittoria Poli, Valeria Berno, Riccardo L. Rossi, Rolland Reinbold, Max Endele, Timm Schroeder, Marina Rocchigiani, Żaneta Szkarłat, Salvatore Oliviero, Stephen Dalton, Alessio Zippo

**Affiliations:** 1 Department of Epigenetics, Fondazione Istituto Nazionale di Genetica Molecolare ‘Romeo ed Enrica Invernizzi', Via Francesco Sforza 35, Milan 20122, Italy; 2 Institute of Biomedical Technologies, National Research Council, via Cervi 93, Segrate-Milan 20090, Italy; 3 Department of Biosystems Science and Engineering, ETH Zurich, Mattenstrasse 26, Basel 4058, Switzerland; 4 Dipartimento di Biotecnologie, Chimica e Farmacia Università di Siena, Via Fiorentina 1, Siena 53100, Italy; 5 Human Genetic Foundation (HuGeF), via Nizza 52, Torino 10126, Italy; 6 Department of Biochemistry and Molecular Biology, University of Georgia, 500 D.W. Brooks Drive, Athens, Georgia 30602, USA

## Abstract

Stem cell identity depends on the integration of extrinsic and intrinsic signals, which directly influence the maintenance of their epigenetic state. Although Myc transcription factors play a major role in stem cell self-renewal and pluripotency, their integration with signalling pathways and epigenetic regulators remains poorly defined. We addressed this point by profiling the gene expression and epigenetic pattern in ESCs whose growth depends on conditional Myc activity. Here we show that Myc potentiates the Wnt/β-catenin signalling pathway, which cooperates with the transcriptional regulatory network in sustaining ESC self-renewal. Myc activation results in the transcriptional repression of Wnt antagonists through the direct recruitment of PRC2 on these targets. The consequent potentiation of the autocrine Wnt/β-catenin signalling induces the transcriptional activation of the endogenous Myc family members, which in turn activates a Myc-driven self-reinforcing circuit. Thus, our data unravel a Myc-dependent self-propagating epigenetic memory in the maintenance of ESC self-renewal capacity.

## Abstract (web summary)

The Myc transcription factor is a major regulator of stem cell (SC) self-renewal and pluripotency but how this integrates signals from other pathways is unclear. Here, the authors show that Myc activation triggers epigenetic memory in self renewing embryonic SCs via PRC2-mediated potentiation of the Wnt pathway.

## Graphical abstract

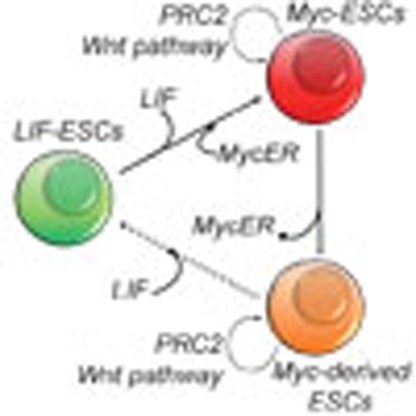

During development, transient signals induce changes in gene expression pattern and chromatin structure, which define cell identity and differentiation potential^1^,^2^. Epigenetic memory plays a central role in the maintenance of cell identity and influences cell responsiveness to environmental cues, thus governing cell plasticity^3^,^4^,^5^. Chromatin regulators and self-reinforcing regulatory transcription networks (TRNs) drive the onset of epigenetic memory, which is then propagated through stem cell self-renewal and somatic cell proliferation^6^. Among them, the Polycomb (PcG) and the Trithorax (TrxG) group of proteins are involved in the maintenance of the repressive and active transcription states, respectively^7^. In embryonic stem cells (ESCs), developmental genes are targeted by both TrxG and PcG complexes, leading to the formation of a permissive chromatin state characterized by the co-existence of H3K4me3 mark embedded in H3K27me3 domains^8^,^9^. The epigenetic state of ESCs is maintained by continuous exposure to signals that converge on chromatin to reinforce the self-propagating TRN^3^,^10^,^11^,^12^,^13^. The transcription factors Oct4, Sox2 and Nanog sustain the ES-specific gene expression programme through an interconnected regulatory loop^14^. Maintenance of ESC self-renewing state relies on exogenous stimulation with leukemia inhibitory factor (LIF) and bone morphogenetic protein 4 (BMP4) growth factors and the consequent activation of their downstream effectors Stat3 and Smad1, which integrate with the core TRN by co-occupying enhancers bound by Oct4, Sox2 and Nanog^11^. More recently, it has been shown that dual inhibition (2i) of Fgf4/MEK/Erk and GSK3-β signalling pathways shields ESCs from autocrine differentiation cues, thus stabilizing a naïve pluripotent ground state^15^. Of importance, the inhibition of GSK3-β reinforces the Wnt/β-catenin signalling, which ultimately counteracts the Tcf3 transcriptional repression activity on the TRN^16^,^17^,^18^. The ESC dependency on LIF/Stat3 signalling could be circumvented by either inhibiting pro-differentiation regulators^15^,^19^,^20^ or by enforcing expression of pluripotency factors^21^,^22^,^23^. Among these, the Myc family members *Myc* and *Mycn* have been described to modulate self-renewal and pluripotency of ESCs. Functionally, the concomitant deletion of both Myc and Mycn in pluripotent stem cells affects self-renewal and induces cell differentiation^23^,^24^,^25^,^26^. At the molecular level, Myc target genes are involved in cell cycle regulation, cell growth and metabolism, thus regulating a distinct subset of genes respect to those targeted by the core pluripotency-associated transcription factors^11^,^27^. Importantly, Myc directly represses genes involved in cell fate specification such as the master regulator Gata6, through poorly defined molecular mechanisms^25^. Despite the proven function of Myc in stem cell self-renewal and pluripotency, its role in maintaining the epigenetic state of ESCs have not been addressed so far.

Here we report a unique role of Myc in sustaining ESC identity, which relies on the potentiation of the Wnt/β-catenin signalling through the PRC2-dependent epigenetic silencing of Wnt antagonists. This regulatory cascade establishes a positive feedback loop by inducing the transcriptional activation of the endogenous *Myc* and *Mycn* genes. Once established, this Myc self-reinforcing circuit is sufficient to trigger an epigenetic memory in ESCs, which in turn self renew in the absence of further extrinsic or intrinsic signals.

## Results

### Myc sustains self-renewal of ESCs

**Figure 1:**
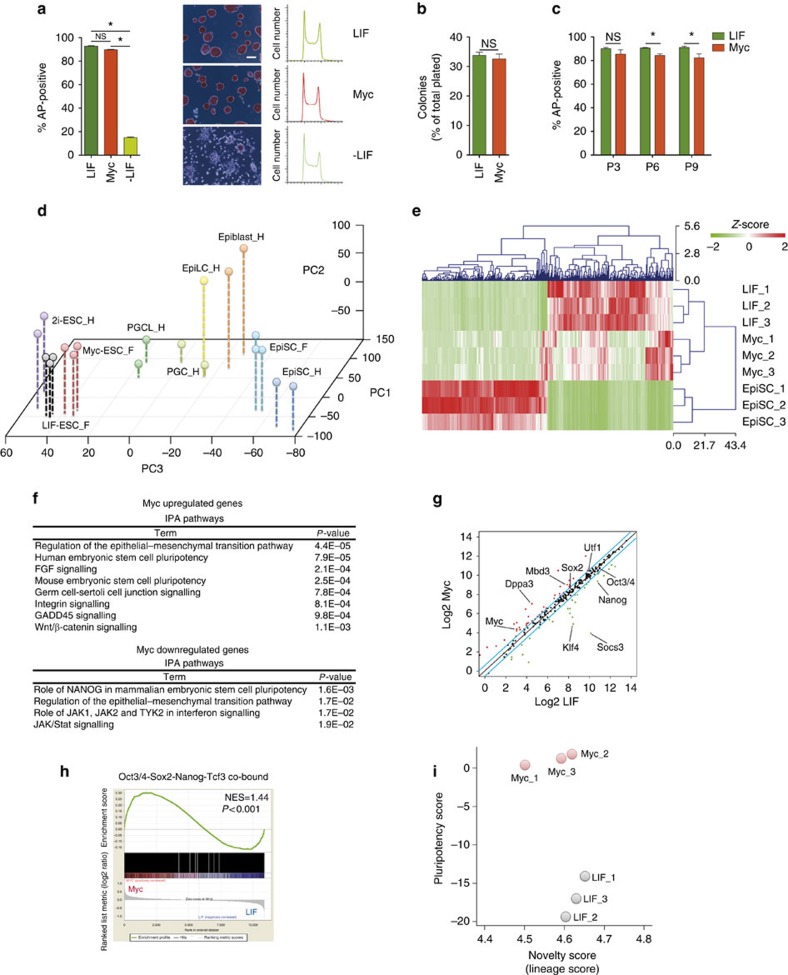
Myc sustains mouse ESCs identity by activating a specific transcriptional programme. (**a**) Alkaline phosphatase (AP) staining of murine MycER ESCs grown for 3 days in the presence of LIF, OHT (Myc) or in -LIF conditions. Relative quantifications of positive colonies are represented as percentage of the total colonies formed. Representative images of stained ESCs maintained for 3 days in the indicated conditions (middle panels, scale bar, 200 μm) and the relative cell cycle profiles (right panels) are shown. (**b**) Single-cell clonogenic assay was performed and the relative number of colonies grown in the indicated conditions was quantified and represented as percentage of the total number of single-cell plated. (**c**) Long-term colony-forming assay was performed culturing cells in the presence of LIF or OHT (Myc) and the relative quantification of AP-positive colonies was assessed at the indicated passages, until 18 days. (**d**) Principal component analysis (PCA) of gene expression profile of ESCs grown in the presence of either LIF (LIF-ESC_F) or OHT (Myc-ESC_F) and EpiSC (EpiSC_F). Each sample is represented in triplicates and the analysis was integrated with previously published data profiling the expression pattern of 2i grown ESCs, EpiLC, EpiSC, epiblasts (E5.75), primordial germ cell-like cells (PGCLCs) and primordial germ cells (PGCs) at E9.5 (ref. 29). (**e**) Heat map of the most differentially expressed genes (cutoff was set to −1.5>fold change>1.5, 200 genes) between LIF-ESCs and EpiSCs. (**f**) Ingenuity pathway analysis (IPA) of differentially regulated genes between LIF- and Myc-dependent ESCs (*n*=3). (**g**) Scatter plot of pluripotency genes (Supplementary Data 1) expression levels, measured in LIF versus Myc-dependent ESCs (*n*=3). (**h**) Gene set enrichment analysis (GSEA) of Oct4/Sox2/Nanog/Tcf3 common target genes in Myc- versus LIF-maintained ESCs (*n*=3). NES=normalized enrichment score. (**i**) Machine-learning classification of gene expression pattern of LIF and Myc-dependent ESCs obtained by applying the PluriTest assay. The PluriTest classifier assessed the pluripotency potential of the examined biological samples on the bases of their gene expression profiles. Data in panels **a**, **b** and **c** are means±s.e.m. (*n*=3). (*P<0.05; NS, not significant; Student's *t*-test). See also related Supplementary Figs 1–3.

To determine the functional role of Myc on the maintenance of murine ES cells identity, we compared ESCs grown either in LIF-containing media or in a Myc-dependent manner (Fig. 1a). To this purpose, we took advantage of ES Myc^T58A^ER cells (thereafter named MycER)^23^ expressing an exogenous MycER fusion protein activated by 4-hydroxytamoxifen (OHT). Myc-dependent ESCs (Myc), which were maintained in the absence of LIF and in the presence of OHT stimulation, behaved similar to the LIF grown cells with respect to numbers of dome-shaped and alkaline phosphatase positive (AP+) colonies, in accordance with previously reported data^23^ (Fig. 1a; Supplementary Movies 1 and 2). Similar results were obtained both in single-cell and in long-term self-renewing assays (Fig. 1b,c). These data suggest that MycER activation can replace LIF signalling in the long-term maintenance of ESCs. Considering that Myc plays a major role on cell cycle control, we tested whether Myc ability to promote ESCs self-renewal was due to altered cell cycle and/or proliferation in MycER cells. The cell cycle profile, the rate of proliferation and cell division were comparable between LIF-maintained and Myc-dependent ESCs (Fig. 1a and Supplementary Fig. 1a,b). Accordingly, single-cell tracking analyses show similar timing and pattern of cell divisions of LIF- and Myc-ESCs, which are characterized by symmetric divisions, in agreement with their self-renewing potential (Supplementary Fig. 1b and Supplementary Movies 1 and 2). These results suggested that perturbations of cell proliferation and/or cell cycle progression could not account for Myc-dependent maintenance of ESC identity. To exclude that the above results could be ES Myc^T58A^ER clone dependent, we generated an independent R1 ESC clone (named A2), expressing similar level of the exogenous MycER (Supplementary Fig. 1c) and we obtained comparable results (Supplementary Fig. 1d–f). Taken together, these data suggest that MycER activation is sufficient to maintain ESCs identity through a LIF-independent regulatory circuit.

### Myc activates an alternative transcription programme in ESCs

To address the molecular mechanisms through which Myc supports ESC identity, we performed gene expression profile analyses of Myc- and LIF-maintained ESCs, together with epiblast stem cell (EpiSC), as a control for a primed state of stemness. Integrated principal component analysis (PCA) showed that Myc-ESCs clustered together with LIF-ESCs and were distant from both EpiSC and primordial germ cells^28^,^29^ (Fig. 1d and Supplementary Fig. 2a). Despite these similarities, a specific gene expression signature distinguished the Myc- from the LIF-ESCs (Fig. 1e,f and Supplementary Fig. 2b). Ingenuity pathway analysis (IPA) indicated that pluripotency-associated genes were enriched among Myc upregulated genes while Nanog-correlated genes were downregulated. This agrees with the fact that the Nanog transcript, together with other LIF/Stat3 target genes^22^,^30^, were downregulated in Myc- respect to LIF-ESCs (Fig. 1f,g and Supplementary Fig. 2c). At the same time, a subset of pluripotency transcription regulators^31^ including Dppa3, Utf1, Nr0b1 and Myc were upregulated in Myc-ESCs, whereas Oct4 and Sox2 were unchanged (Fig. 1g and Supplementary Fig. 2c). Importantly, gene set enrichment analysis (GSEA) revealed that the Oct4/Sox2/Nanog/Tfc3 co-bound targets^32^ were enriched in Myc- respect to LIF-ESCs (Fig. 1h). In addition, the machine-learning classifier PluriTest^33^ highlighted a relative enhancement of pluripotency features in Myc-ESCs (Fig. 1i). Altogether, these multiple bioinformatic approaches indicate that Myc potentiates pluripotency-associated genes in ESCs. In agreement with these findings, flow cytometry analysis of Nanog and Oct4 shows how LIF- and Myc-dependent ESCs are homogeneous cell populations, with respect to heterogeneous ESCs grown in the absence of LIF (-LIF; Supplementary Fig. 2d). Finally, GSEA and functional annotation clustering showed that signatures linked to developmental processes were over-represented in LIF-ESCs. These findings were further supported by the observation that developmental genes, which are targeted and repressed by Polycomb proteins Ring1b (PRC1) and Eed (PRC2) were downregulated in Myc-ESCs^34^ (Supplementary Fig. 2e–h). In addition, genome-wide expression profile of *Eed* knock-downed ESCs revealed a strong correlation between Myc downregulated and Eed-repressed genes (Supplementary Fig. 2i). To determine the direct contribution of Myc in activating this alternative transcriptional programme supporting ESC maintenance, we profiled gene expression of ESCs grown in the presence of both LIF and OHT (LIF+Myc-ESCs) and compare it to the profiles of LIF- and Myc-ESCs. The obtained results further confirmed that Myc supports the transcriptional activation of pluripotency-associated genes in ESCs (Supplementary Fig. 3a–c). Importantly, LIF-, LIF+Myc- and Myc-ESCs showed equivalent total RNA and mRNA levels indicating that global transcriptional amplification^35^,^36^ did not occurred in this setting (Supplementary Fig. 3d), as observed upon MycER activation in proliferating fibroblasts^37^. Collectively, the above data suggest that Myc supports the self-renewal of ESCs by activating a LIF-independent alternative transcriptional regulatory programme, reinforcing the TRN.

### Myc requires Wnt/β-catenin pathway to maintain ESCs

**Figure 2:**
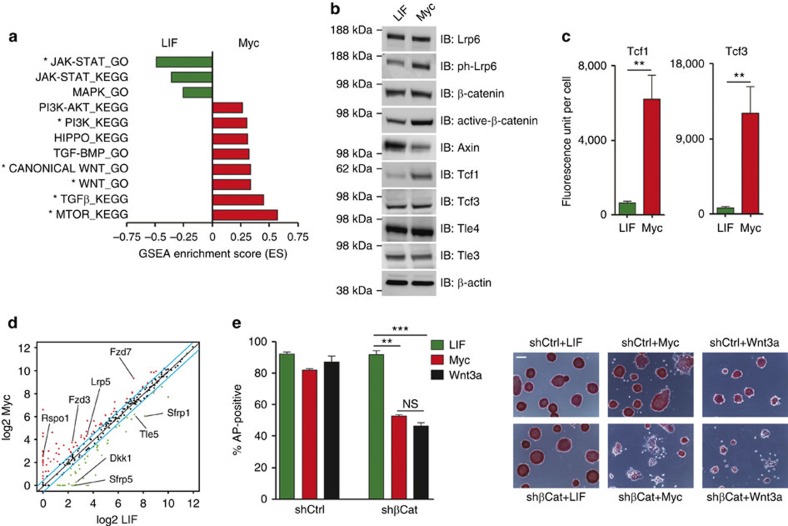
Myc requires the Wnt/β-catenin signalling to sustain ESC self-renewal. (**a**) Gene set enrichment analysis (GSEA) of signalling associated gene sets (Supplementary Data 1) in Myc- versus LIF-maintained ESCs (*n*=3; *P<0.0001). NES=normalized enrichment score. (**b**) Western blot analysis of Wnt/β-catenin signalling pathway-related proteins. Total protein extracts were collected from LIF- and Myc-dependent ESCs, and immunostaining (IB) analysis was performed using the indicated antibodies; β-actin was used as loading control. (**c**) *In situ* proximity ligation assay (PLA) was performed to measure the proximity between Tcf1 or Tcf3 and β-catenin within cell nuclei of LIF- and Myc-dependent ESCs. (**d**) Scatter plot of expression level of Wnt pathway-associated genes (Supplementary Data 1) measured in LIF- versus Myc-dependent ESCs (*n*=3). (**e**) AP staining of MycER ES cell clones expressing either a control shRNA (shCtrl) or a β-catenin shRNAs (shβCat) and grown for 3 days in the indicated conditions; relative quantification of positive colonies are represented as percentage of the total colonies formed. Representative images of stained ESCs grown in the different conditions are shown. Scale bar, 200 μm. Data in panels **c** and **e** are means±s.e.m. (*n*=3). (**P<0.01, ***P<0.001; NS, not significant; Student's *t*-test). See also related Supplementary Fig. 4.

We next examined whether Myc could reinforce the TRN by modulating signalling involved in pluripotency^15^,^17^,^38^. Both IPA and GSEA identified the Jak/Stat3 and the MAPK pathways being over-represented in LIF-ESCs^39^,^40^, whereas Myc potentiates the Wnt/β-catenin signalling (Figs 1f and 2a and Supplementary Fig. 4a and b). Importantly, the direct comparison of the gene expression profile of LIF-, Myc- and LIF+Myc-ESCs further confirmed that Myc drove the reinforcement of Wnt/β-catenin signalling, whereas the LIF withdrawal caused the downregulation of the Jak/Stat3 pathway (Supplementary Fig. 3b-c).

To understand how Myc sustains the Wnt pathway, we first performed protein level analyses, showing that this Myc-dependent potentiation occurred at multiple stages (Fig. 2b). At the cell membrane level, we showed the activation of the Fzd/Lrp receptor complex resulting in the increased phosphorylation of Lrp6 in Myc-ESCs^41^. At the level of the destruction complex, which promotes β-catenin degradation, we registered reduction of the rate-limiting factor Axin1 and inhibition of GSK3β^41^,^42^,^43^ (Fig. 2b and Supplementary Fig. 4c). Interestingly, the latter could be achieved by Myc via AKT-mediated phosphorylation of GSK3β (Fig. 2b and Supplementary Fig. 4c). In addition, we found that the activation of the Wnt pathway in Myc-ESC determined the accumulation of active β-catenin (Fig. 2b) and its association with the downstream effectors Tcf1 and Tcf3 in ESCs nuclei, as measured by a proximity ligation assay (PLA; Fig. 2c and Supplementary Fig. 4d,e)^16^,^17^. At the transcriptional level, we observed that while genes coding for Wnt receptors and co-receptors were upregulated, antagonists of the Wnt pathway, such as the Dkk and Sfrp family members, were downregulated in Myc-dependent ESCs, with respect to LIF-ESCs (Fig. 2d and Supplementary Fig. 4f).

Thereafter, we determined whether the activation of the MycER protein recapitulates the physiological role of endogenous Myc proteins in sustaining Wnt signalling pathway in ESCs. We transduced MycER ESCs with lentivirus expressing inducible small hairpin RNA (shRNA) for both Myc and Mycn (i-dKD), thus allowing us to tightly control the level and the timing of gene silencing (Supplementary Fig. 4g). We observed that knocking down endogenous Myc proteins results in reduced Wnt-pathway activation, as highlighted by less active β-catenin in the i-dKD ESCs, which is caused by the modulation of both the responsiveness to the autocrine Wnt pathway, and the deconstruction complex activity (Supplementary Fig. 4g). Altogether, the obtained results indicated that Myc reinforced the Wnt signalling pathway in ESCs.

We then investigated whether the capacity of Myc in maintaining ESCs depends on the autocrine Wnt signalling. We found that knocking-down β-catenin strongly affected self-renewal of Myc-dependent ESCs, whereas it did not perturb ESCs growth in LIF dependency (Fig. 2e and Supplementary Fig. 4h)^16^,^18^. In addition, we showed that treating Myc-ESCs with soluble Wnt inhibitors Dkk1 and sFRP1 induced loss of pluripotency. Importantly, these effects were counteracted by stimulating ESCs with soluble Wnt3a (Supplementary Fig. 4i)^38^.

Taken together, these data suggest that Myc exerts its function on ESCs self-renewal by reinforcing the Wnt/β-catenin signalling via transcriptional modulation of Wnt genes.

### Myc alters the epigenetic state of bivalent genes in ESCs

**Figure 3:**
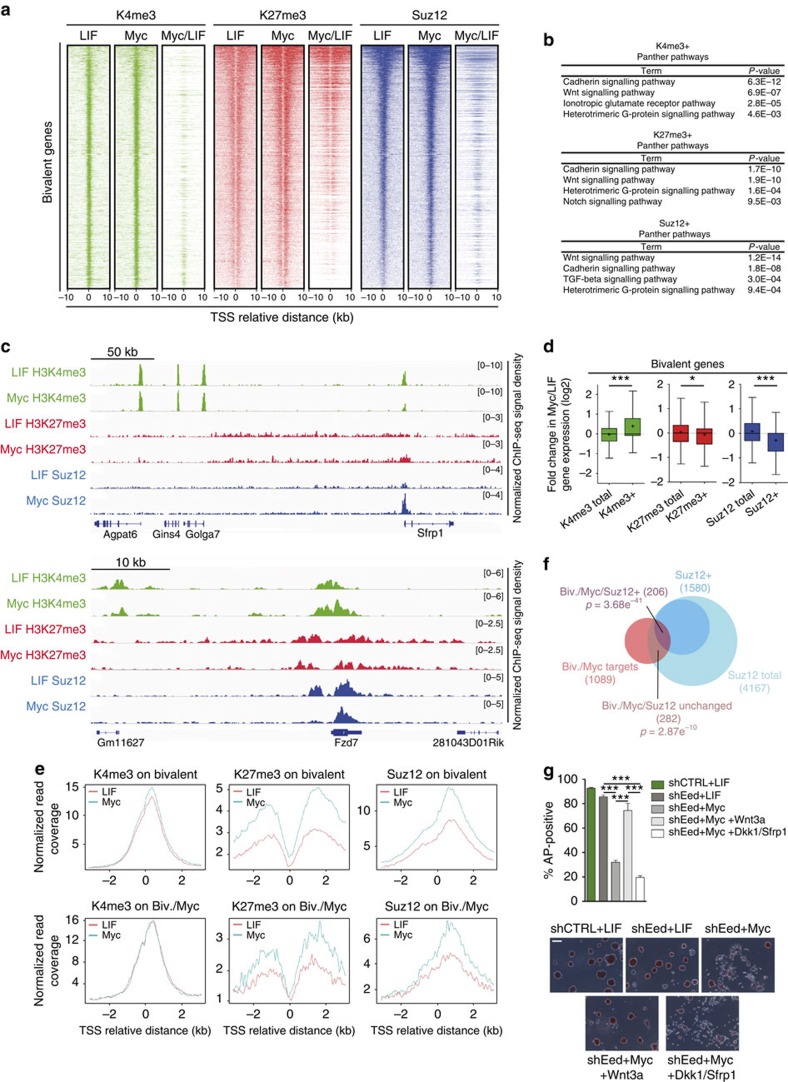
Myc alters the epigenetic state of bivalent genes. (**a**) Heat maps of normalized read density of H3K4me3 and H3K27me3 deposition and Suz12 binding on bivalent genes in LIF- and Myc-dependent ESCs, ranked according to Suz12 binding in LIF-ESCs. The normalized read coverage against library size was calculated in the distance of ±10 kb to transcriptional start site (TSS) with 50 bp bin. Myc/LIF columns report TSS with read intensity fold-change >2 between Myc- and LIF-ESCs. (**b**) Gene ontology of bivalent genes, which gained either H3K4me3,H3K27me3 or Suz12 in Myc-ESCs, showing relative enriched pathways. (**c**) Genomic snapshots showing H3K4me3, H3K27me3 and Suz12 binding in LIF- and Myc-ESCs on an antagonist (Sfrp1) and an agonist (Fzd7) of the Wnt/β-catenin pathway. The *x* axis corresponds to genomic location; the *y* axis corresponds to ChIP-seq signal density normalized to sequencing depth. (**d**) Box-and-whisker plots of the Myc/LIF gene expression fold-change of bivalent genes, which gained either H3K4me3, H3K27me3 or Suz12 in Myc-ESCs, compared with total bivalent genes marked by H3K4me3, H3K27me3 or Suz12 binding. Boxes encompass the 25th to 75th percentiles. Whiskers extend to 10th and 90th percentiles. The central horizontal bar indicates median fold change, the black cross indicates the mean. (**e**) Histograms showing the average profile of H3K4me3, H3K27me3 and Suz12 in LIF- and Myc-ESCs, centred on TSS of total bivalent genes (up) or only the bivalent genes, which are also Myc(s) targets (down). Window size is ±3 kb, bin size10 bp. (**f**) Overlap between bivalent genes, which are Myc(s) targets (Biv./Myc target), total Suz12 targets (Suz12 total) and genes which gained Suz12 in Myc-ESCs (Suz12+). The *P*-values relative to the hypergeometric probabilities of Biv./Myc targets, which either gained Suz12 in Myc-ESC or not are reported; total genes=22,592. (**g**) AP staining of MycER ESCs expressing either a control (shCtrl) or Eed (shEed) shRNA, grown 3 days in the indicated conditions; relative quantification of positive colonies is represented as percentage of total colonies formed. Representative images are shown. Scale bar, 200 μm. Data in panels **d** and **g** are means±s.e.m. (*n*=3). (*P<0.05, ***P<0.001; Student's *t*-test). See also related Supplementary Fig. 5.

Considering that Wnt pathway-related genes are targets of both PcG and MLL complexes in ESCs^8^,^34^,^44^, we asked whether their transcriptional regulation could depend on Myc-driven epigenetic changes at their bivalent promoters. To this end, we compared the chromatin state between LIF and Myc-ESCs by performing chromatin immunoprecipitation (ChIP)-sequencing experiments using antibodies recognizing H3K4me3, H3K27me3 and the PRC2 component Suz12. Although Myc targets positively correlate with active genes^11^,^27^, we did not measure major changes in the genome-wide pattern of H3K4me3 (Supplementary Fig. 5a). Rather, when we focused our analyses on bivalent genes, we observed a redistribution of this histone mark with a relative increased level on PRC2-low-bound genes in Myc-ESCs (Fig. 3a,e). Genome-wide mapping of H3K27me3 showed an increased enrichment specifically at bivalent genes promoters (Fig. 3a,e and Supplementary Fig. 5a–c). Among the K27 gained regions, we measured a relative enrichment at those genes, which are target of both PRC2 and PRC1 complexes and are characterized by large PcG domains^44^ (Fig. 3a and Supplementary Fig. 5c). This pattern was mirrored by an augmented binding of Suz12 at bivalent genes with a preferential association with PRC1 targets (Fig. 3a,e and Supplementary Fig. 5c). Of note, we observed that some active genes related to the Jak/Stat3 pathway gained H3K27me3, thus leading to the formation of *de novo* bivalent genes in Myc-ESCs (for example, *Socs3* gene in Supplementary Fig. 5e). IPA indicated that both K4 (PRC2-low) and K27/Suz12 gained regions are enriched for Wnt pathway-related genes with the former containing genes coding for receptors and co-receptors (for example, *Fzd7*), whereas the latter being enriched for antagonists (for example, *Sfrp1*; Fig. 3b,c). We then correlated modulation in gene expression with the measured changes in chromatin features. The obtained results showed that chromatin perturbations in Myc-ESCs positively correlated with the transcriptional pattern of the targets, with the up- and downregulated genes being enriched for either H3K4me3 or H3K27me3 and Suz12 binding, respectively (Fig. 3d and Supplementary Fig. 5d). We then determined the direct contribution of Myc binding to the newly defined chromatin landscape by measuring the relative enrichment of K4me3, K27me3 and Suz12 at bivalent genes, which are directly bound by Myc or Mycn in ESCs (Fig. 3e-f). This analysis showed that among the Myc targets, H3K4me3 is unchanged, whereas both K27me3 and Suz12 binding are strongly enriched, thus suggesting a Myc-dependent modulation of PRC2 binding.

Considering the discovered role of PRC2 in controlling the epigenetic state of Wnt pathway-related genes, we analysed the effects of either Eed or Ezh2 knockdown on Myc-ESCs self-renewal capacity (Fig. 3g and Supplementary Fig. 5f–i). We observed that Myc activation, in the absence of the PRC2 complex, was not sufficient to maintain ESC self-renewal as shown by the reduced number of dome-shaped and AP+ colonies (Fig. 3g and Supplementary Fig. 5i). Importantly, the PRC2-dependent impairment of Myc-ESC self-renewal was restored upon reactivation of Wnt/β-catenin signalling by treating cells with Wnt3a (Fig. 3g and Supplementary Fig. 5i). Collectively, the obtained results showed that Myc determines an alternative epigenetic state at subsets of bivalent genes, which triggers the transcriptional repression of Wnt antagonists.

### Myc directly recruits PRC2 at Wnt antagonist genes

**Figure 4:**
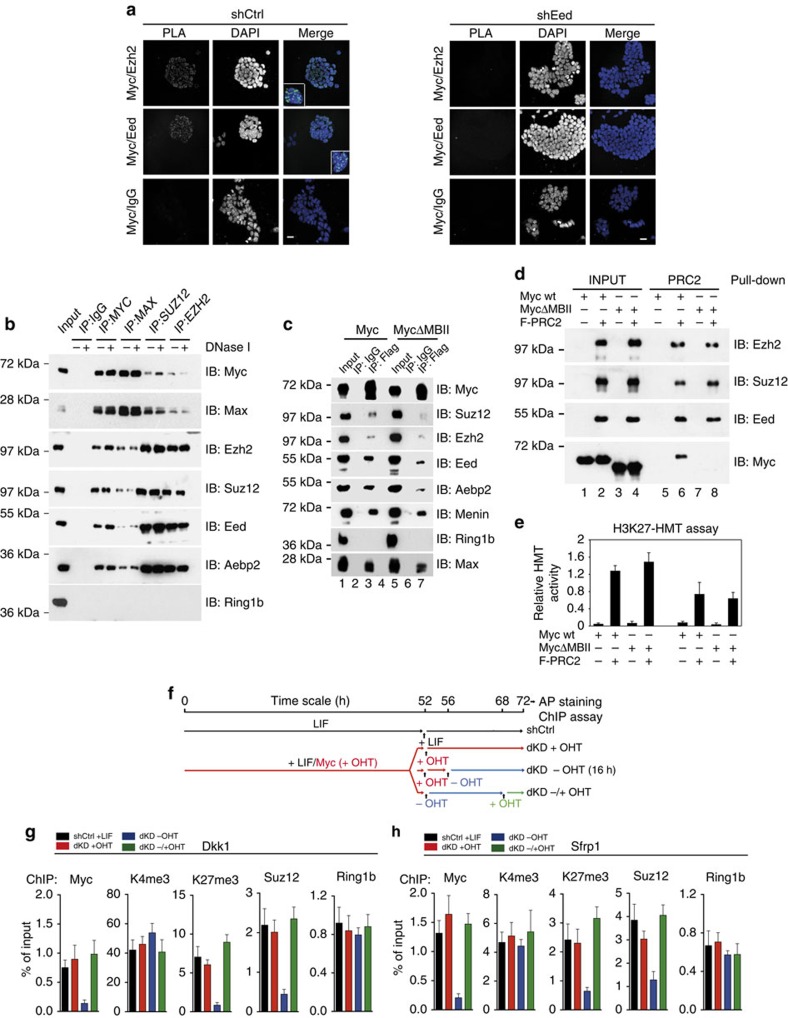
Myc represses Wnt pathway antagonists via PRC2. (**a**) *In situ* proximity ligation assay between Ezh2 or Eed and Myc, in LIF-ESCs, expressing either a control (shCtrl) or an Eed (shEed) shRNA. The proximity between Myc and an unrelated protein (IgG) was assessed as negative control. Scale bar, 50 μm. (**b**) Nuclear extracts obtained from ESCs were either immunostained (Input) or subjected to immunoprecipitation (IP) with indicated antibodies in the presence or absence of DNaseI. Immunostaining (IB) was performed using the indicated antibodies; 10% of the total protein samples were loaded as input. (**c**) Nuclear extracts from HEK 293 cell clones expressing the Flag-Myc or the Flag-MycΔMBII were used to immunoprecipitate. Myc proteins with anti-Flag antibody and interacting proteins were revealed by immunostaining using the indicated antibodies. (**d**) Recombinant PRC2 complex was used to pull-down the His-Myc wt or the His-MycΔMBII recombinant proteins. The PRC2 complex was bound to the Flag-resin and the Myc protein interactions were measured by immunostaining after peptide competition elution. (**e**) H3K27-histone methyltransferase activities (HMT) of the eluted protein complexes were measured by a colorimetric assay. (**f**) Schematic representation of the experiment: the control (shCtrl) and the double c- and N-Myc (dKD) ESCs were maintained, respectively, in LIF (black line) or in LIF+OHT (red line). At the indicated time point, the dKD ESCs were either grown in the presence of OHT (+OHT, red line) or shifted to LIF only medium (−OHT, blue line) for the following 16 h. Thereafter, the MycER protein was reactivated upon 4 h treatment with OHT (−/+ OHT, green line). (**g**,**h**) ChIP onMycER ESCs grown in the presence of LIF and expressing either a control (shCtrl) or Myc and Mycn (dKD) shRNAs. The levels of K4me3, K27me3, Myc, Suz12 and Ring1b at the TSS of the indicated genes was measured in the control cells (black bars), in the dKD cells maintained in the presence of OHT (red bars) or after OHT withdrawal for 16 h (blue bars), followed by the re-induction of MycER fusion protein by OHT treatment for additional 4 h (green bars). Data in panels **g** and **h** are means±s.d. (*n*=3). See also related Supplementary Figs 6 and 7.

To further determine the direct contribution of Myc to the enrichment of H3K27me3 at Wnt antagonist genes, we investigated a possible association between endogenous Myc and PRC2. PLAs showed that within the cellular context, both Myc and Mycn associated with PRC2 components Ezh2 and Eed in multiple foci (Fig. 4a and Supplementary Fig. 6a–d). Importantly, the specificity of the defined associations was further validated by knocking-down either Myc transcription factors or Eed (Fig. 4a and Supplementary Fig. 6a–d). By performing Co-IP experiments, we found that endogenous Myc associated with PRC2 core complex (Eed, Suz12 and Ezh2) and the associated Aebp2 protein, but not with PRC1 core component Ring1B (Fig. 4b). We then assessed the binding affinity and the kinetics of PRC2–Myc protein interactions by immunoprecipitating Myc from ESC nuclear extracts with increased salt concentrations. We observed that the Myc–PRC2 complex is stable up to 200 mM NaCl/1% Triton, and therefore showed an intermediate binding affinity compared with known Myc-associated proteins Tip60 and Baf53a (Supplementary Fig. 6e)^27^. After enriching for the Myc-interacting protein complexes using an affinity chromatography purification approach, we separated the eluted interactors by gel filtration and showed that Myc associated with an intact and enzymatically active PRC2 complex (Supplementary Fig. 6f–g). Mutagenesis experiments indicated that the conserved MBII motif, located in the Myc transactivation domain contributed in mediating the MYC–PRC2 interaction (Fig. 4c). By using *in vitro* reconstituted PRC2 complex and recombinant Myc proteins, we found that Myc interacted directly with an enzymatically active PRC2 complex in an MBII-dependent manner (Fig. 4d-e). By performing GST-Pull down assay between Myc and each PRC2 components, we showed that Eed and the cofactor Aebp2 mediated the direct association with Myc *in vitro* (Supplementary Fig. 6h–i). To demonstrate the direct interaction between Myc and PRC2 within the cellular context, we performed acceptor photobleaching resonance energy transfer (apFRET) assays (Supplementary Fig. 6j–k). The PRC2 proteins Eed and Aebp2 were fused with the cyan fluorescent protein (CFP), whereas Myc wt and MycΔMBII were fused to the yellow fluorescent protein (YFP) and co-expressed in 3T3 cells. apFRET showed that both Eed and Aebp2 interacted with Myc, whereas their co-expression with MycΔMBII resulted in a reduction of their molecular interactions (Supplementary Fig. 6j,k). Altogether, this set of data demonstrates the direct interaction of both endogenous Myc and Mycn with the PRC2.

Next, we investigated the possibility that Myc could recruit PRC2 to Wnt antagonist genes. To this end, we knocked-down both endogenous Myc and Mycn in MycER ESCs (dKD), thus being able to modulate Myc protein levels upon OHT treatment (Supplementary Fig. 7a,b). The dKD ESCs behaved similar to the control cells (shCtrl) as far as self-renewal capacity, doubling time and cell cycle profile were concerned, whereas a prolonged withdrawal of OHT (72 h) was not compatible with the maintenance of self-renewal capacity (Supplementary Fig. 7c–e)^24^,^25^. Of importance, we determined that a short withdrawal of OHT (16 h) did not affect ESC self-renewal, thus permitting to measure the direct effects of Myc inactivation on chromatin states (Supplementary Fig. 7c–e). We performed ChIP assay in ESCs in which we modulated Myc protein levels and we found that PRC2 binding and H3K27me3 level on the promoter of genes encoding for Wnt antagonists (*Dkk1*, *Sfrp1*, *Sfrp5* and *Apccd1*) were strongly reduced upon short OHT withdrawal and rescued by MycER re-activation (Fig. 4f–h and Supplementary Fig. 7f,g). Ring1B binding was not affected by Myc inactivation or by its rescue, suggesting a H3K27me3-independent mechanisms of PRC1 recruitment on these loci^34^,^45^ (Fig. 4g,h). Of note, in the same experimental conditions, other bivalent genes (*Sox17* and *Fzd1*) did not show this dependence upon Myc activation (Supplementary Fig. 7h,i). Taken together, these results indicate that Myc, by controlling the H3K27me3 state at Wnt antagonist genes through the recruitment of the PRC2 complex, reinforces the autocrine Wnt signalling pathway, thereby promoting ESC pluripotency.

### Myc sustains a self-reinforcing positive feedback loop

**Figure 5:**
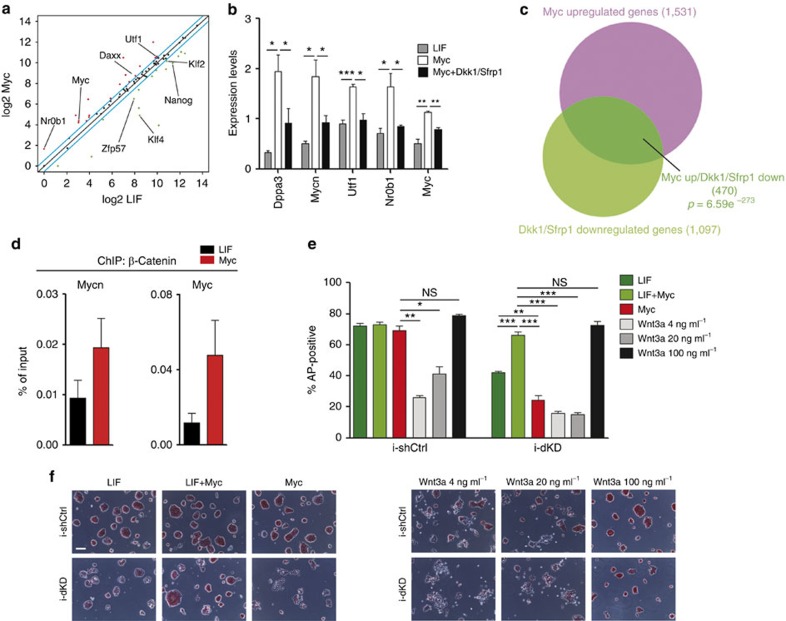
Myc sustains a self-reinforcing positive feedback loop. (**a**) Scatter Plot of the expression level of pluripotency and Tcf3-target genes (Supplementary Data 1), measured in LIF versus Myc-dependent ESCs (*n*=3). Transcript levels are shown in log2 scale. (**b**) The relative transcriptional level of a set of the Wnt downstream target genes was confirmed by qRT–PCR analysis on RNAs extracted from ESCs grown in the indicated conditions for 3 days. (**c**) Venn diagram representing the overlap between Myc upregulated and Dkk1/sFRP1 downregulated genes in ESCs. The *P*-value relative to the hypergeometric probability of Myc upregulated genes, which are downregulated by Dkk1/sFRP1 treatment is reported; total genes=22,592. (**d**) The β-catenin binding at the Wnt responsive element (WRE) of both *Myc* and *Mycn* genes was measured by ChIP assay in LIF- and Myc-ESCs. (**e**) Alkaline phosphatase (AP) staining of MycER ES cell expressing either an IPTG-inducible control (shCtrl) or an IPTG-inducible Myc and Mycn shRNAs (dKD) and grown in the indicated conditions for 3 days; relative quantification of positive colonies are represented as percentage of the total colonies formed. (**f**) Representative images of stained ESCs grown in the different conditions are shown. Scale bar, 200 μm. Data in panels **b**, **d** and **e** are means±s.e.m. (*n*=3). (*P<0.05, **P<0.01, ***P<0.001; NS, not significant; Student's *t*-test).

To identify the downstream effectors of Wnt pathway required for Myc-ESCs maintenance, we crossed the geneset of pluripotency factors^31^ with the co-bound Oct4/Sox2/Nanog and Tcf3 targets^32^ (Supplementary Data 1) and analysed their expression profile in Myc- versus LIF-ESCs (Fig. 5a). We found that treatment of Myc-ESCs with soluble Dkk1/sFRP1 inhibited the MycER-dependent transcription activation of endogenous Myc and Mycn, suggesting that their regulation depends on the MycER-driven reinforcement of Wnt signalling (Fig. 5b). Importantly, this effect was not restricted to the analysed genes as global gene expression profiles showed that 30% of Myc-ESC upregulated genes are downregulated in Dkk1/sFRP1-treated cells, meaning that they are downstream of the Wnt pathway (Fig. 5c). Moreover by ChIP assay, we found that in Myc-ESCs, higher amount of β-catenin was associated with the Wnt responsive element of both Myc and Mycn genes (Fig. 5d).

Thereafter, we evaluated whether endogenous Myc proteins are required to sustain Myc-ESC self-renewal capacity. Again, we made use of the inducible Myc and Mycn double knockdown ESCs (i-dKD, Supplementary Fig. 4g) and we observed that the reduction of ∼50% of the total amount of Myc proteins was sufficient to block the MycER-induced self-renewing circuit in Myc-ESCs (Fig. 5e,f). The role of endogenous Myc(s) as downstream targets of the Wnt pathway was further illustrated by the finding that while control ESCs responded in a dose-dependent manner to Wnt3a-induced self-renewal, the i-dKD cells did not (Fig. 5e,f). Taken together, these findings showed that the activation of MycER established a positive feedback loop by sustaining the Wnt signalling, which in turn triggers the transcriptional activation of the endogenous *Myc* and *Mycn* genes. This self-reinforcing circuit plays a major role in maintaining the identity of Myc-dependent ESCs.

### The Myc-driven self-reinforcing circuit maintains ESCs

**Figure 6:**
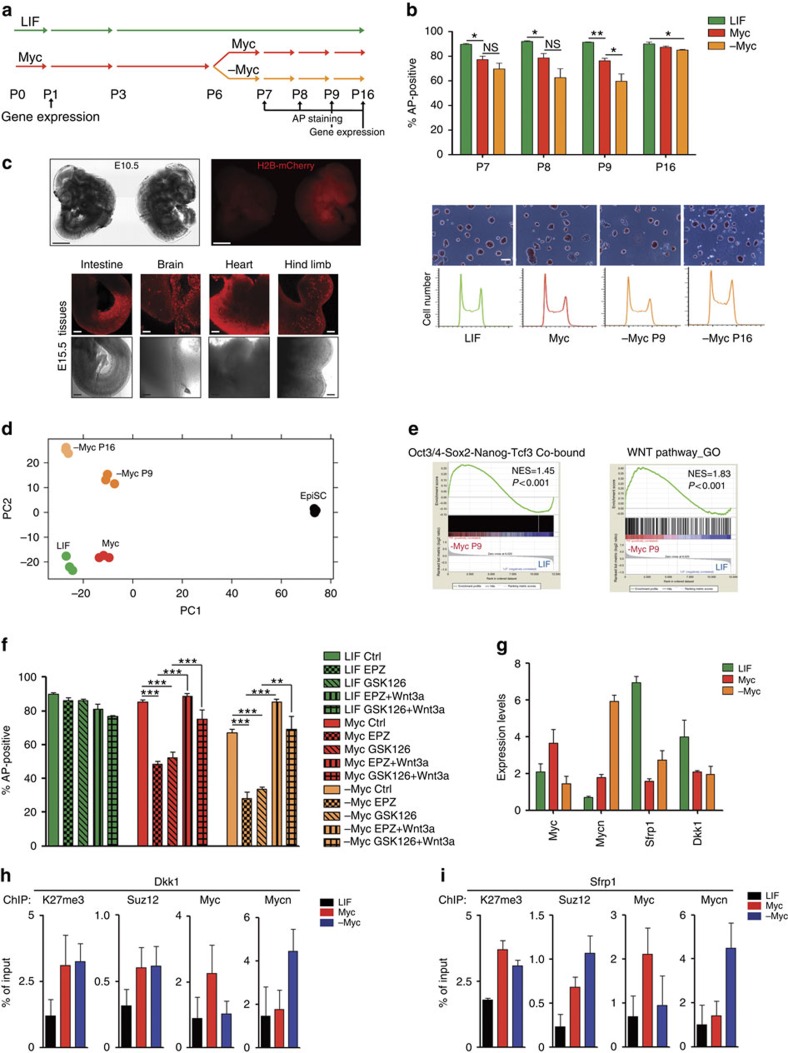
Myc-derived ESC acquired a PRC2-dependent epigenetic memory. (**a**) Schematic representation of the experiment. R1 MycER cells were grown either in the presence of LIF (green line) or in the absence of LIF and in the presence of OHT (Myc, red line). Myc-ESCs were grown for six passages in Myc-dependency (+OHT) and then were maintained in either the same culture condition (Myc) or in the absence of both OHT and LIF (Myc-derived ESCs and indicated in figures as -Myc, orange line in panel). Alkaline phosphatase (AP) activity and gene expression profiles were analysed at indicated time points. (**b**) AP staining of ESCs at indicated passages and conditions. Representative images and relative cell cycle profiles are shown. Scale bar, 200 μm. (**c**) Fluorescence images (right panel) of a chimeric embryo at E10.5, derived from blastocysts injected with late-passages (P16) Myc-derived ESCs carrying a constitutively active H2B-mCherry transgene (right). A non-chimeric embryo is shown as non-fluorescent control (left). Relative bright-field images are shown on the left; scale bar, 1 mm. Lower panels show fluorescence images of organ tissues dissected from E15.5 chimeric embryos; scale bar, 100 μm (**d**). Principal component analysis (PCA) of triplicate gene expression profiles of LIF-, Myc- and -Myc-ESCs at passages 9 and 16.. Expression profiles of EpiSC are included. (**e**) Gene set enrichment analysis (GSEA) of Oct4/Sox2/Nanog/Tcf3 common targets (left) and Wnt pathway genes (right) in Myc-derived at passage 9 versus LIF-maintained ESCs (*n*=3). NES=normalized enrichment score. (**f**) AP staining of MycER ESCs grown 3 days in indicated conditions. EPZ and GSK126 are chemicalinhibitor of the enzymatic activity of PRC2. (**g**) Relative transcriptional levels of indicated genes in the LIF-, Myc- or Myc-derived ESCs (-Myc). (**h**,**i**) ChIP on MycER ESCs maintained in LIF- or Myc-dependency or the Myc-derived ESCs (-Myc). The level of H3K27me3 and the binding of Myc, Mycn and Suz12 at the TSS of the indicated genes were measured. Data in panels **b** and **f–i** are means±s.e.m. (*n*=3). (*P<0.05; **P<0.01, ***P<0.001; NS, not significant; Student's *t*-test). See also related Supplementary Figs 8–10.

We then investigated whether the Myc-driven self-reinforcing circuit could support the maintenance of ES cell identity in the absence of the originating stimulus, namely the MycER activation^3^. To this end, we derived ESCs from Myc-dependent cells, following MycER inactivation upon withdrawal of OHT (Fig. 6a) and we measured their self-renewal potential and pluripotency. We found that, despite a slight reduction in the number of AP+ colonies in the first passages, these newly established ESCs (thereafter named Myc-derived ESCs and indicated as -Myc in the figures) could be continuously propagated in culture for more than 30 passages in the absence of MycER activation and LIF stimulation (Fig. 6b). Single-cell tracking analyses indicate that the initial reduction of colonies in Myc-derived ESCs (-Myc) is attributable to increased cell death, whereas their rate of proliferation, timing of division, cell cycle profile and cell dimensions were comparable to LIF or Myc-dependent ESCs (Fig. 6b, Supplementary Fig. 8a,b and Supplementary Movies 3–5). In addition, the protein level of pluripotency markers was similar among LIF-maintained, Myc-dependent and Myc-derived cells. (Supplementary Fig. 8c-d). Of note, the maintenance of Myc-derived ESCs was not dependent on the culture condition used to establish them as they could be propagated for several passages also in 2i medium (Supplementary Fig. 8e,f). To verify that the capacity to exit pluripotency was not altered in Myc-derived ESCs, we finely determined the onset of epiblast lineage commitment (Supplementary Fig. 8e)^29^. The obtained results showed that the timing of differentiation towards epiblast-like cells and the gene expression pattern of markers of pluripotency and epiblast cells were similar among LIF-maintained, Myc-dependent and Myc-derived cells (Supplementary Fig. 8g,h). Importantly, Myc-derived ESCs showed hallmarks of pluripotency and they formed teratomas once injected in *Nude* mice, differentiating into the three germ layers (Supplementary Fig. 8i–k). To assess whether Myc-derived ESCs were endowed with naïve pluripotent potential^46^, we determined their capacity to form chimeric embryos, once injected into the blastocysts. We observed that mCherry-labelled Myc-derived ESCs contributed extensively (53%) to the formation of developing embryos, with a high degree of chimerism in multiple tissues, as observed at late gestation embryonic stages (Fig. 6c). Importantly, we ascertained that maintenance of Myc-derived ESCs was not caused by spurious activation of MycER. Specifically, we showed that MycER fusion protein was excluded from the nuclear compartment in Myc-derived ESCs and it was not bound to the chromatin of Myc targets (Supplementary Fig. 9a–c). Accordingly, spurious activation of MycER could not account for derivation and maintenance of Myc-derived ESCs as either suboptimal concentration or short-term exposure to OHT were not compatible with ESCs self-renewal (Supplementary Fig. 9d–g). Although the Myc-derived ESCs do not rely on MycER activation for self-renewing, we asked whether they still depend on the downstream effectors of the self-reinforcing circuit that has been established in the parental Myc-ESCs. We observed that silencing of endogenous Myc and Mycn transcripts in Myc-derived ESCs strongly affected their self-renewal capacity and led to cell differentiation (Supplementary Fig. 9h-i). Finally, to avoid the possibility of a clone-dependent effect, we also obtained Myc-derived ESCs from the independently generated R1 ESC clone A2 (Supplementary Fig. 9j). Taken together, the above results indicated that a prolonged activation of MycER is sufficient to establish self-reinforcing circuits, which support the maintenance of ES cell identity in the absence of the instructing stimulus.

### Myc-derived ESCs acquire a PRC2-dependent epigenetic memory

We then investigated whether the Myc-derived ESCs relied on the propagation of the same transcriptional and epigenetic programmes activated in Myc-maintained ESCs. PCA analysis showed that the global gene expression pattern of Myc-derived ESCs resulted similar to the parental Myc-ESCs respect to EpiSC (Fig. 6d). Both IPA and GSEA analyses showed that pluripotency- and Wnt pathway-related genes are reinforced in Myc-derived relative to LIF-ESCs, whereas Jak/Stat3-related genes are downregulated, thereby displaying a similar pattern of signalling pathways with respect to their parental Myc-ESCs (Fig. 6e and Supplementary Fig. 10a–c). Importantly, the Myc-induced transcriptional programme is stably maintained as it is preserved at late passages in Myc-derived ESCs (Fig. 6d and Supplementary Fig. 10a–c). Given these finding we asked whether the MycER-triggered epigenetic changes that supported the self-reinforcing regulatory circuit in Myc-ESCs were required to maintain Myc-derived ESCs. To verify this point, we assess the role of PRC2 on the self-renewing capacity of Myc-derived ESCs by using small molecules, which inhibit Ezh2 enzymatic activity (EPZ and GSK126). The obtained results showed that PRC2 inhibition affected maintenance of Myc-derived ESCs and H3K27me3 deposition on Wnt antagonists Dkk1 and Sfrp1 (Fig. 6f and Supplementary Fig. 10d). Importantly, exogenous Wnt3a treatment complemented the PRC2 chemical inhibition and restored the ability of Myc-derived cells to self-renew, suggesting that those cells required the PRC2 complex to activate the Wnt/β-catenin signalling (Fig. 6f). We found that in Myc-derived ESCs the transcription levels of Wnt antagonists Dkk1 and Sfrp1 are maintained repressed similar to Myc-ESCs, suggesting that, despite the absence of OHT stimulation, they are epigenetically silenced (Fig. 6g). To support this finding, we performed ChIP assay on these targets and compared the relative level of Suz12, Myc(s) and H3K27me3 among the LIF-, Myc-maintained and Myc-derived ESCs. We showed that the Wnt antagonists were repressed by the increased Suz12 binding and H3K27me3 deposition in Myc-dependent and Myc-derived ESCs, with respect to LIF-ESC (Fig. 6h,i). Importantly, endogenous Myc proteins bound with higher affinity in Myc-dependent and Myc-derived ESCs, supporting the PRC2 recruitment and H3K27me3 deposition on those loci (Fig. 6h,i). Of note, the global level of histone modifications and PRC2 proteins were unchanged and genomic integrity was preserved in Myc-derived ESCs (Supplementary Fig. 10e,f). Altogether, these data demonstrate that those epigenetic changes that supported the self-reinforcing regulatory circuit in the parental Myc-ESCs were maintained in Myc-derived ESCs.

**Figure 7:**
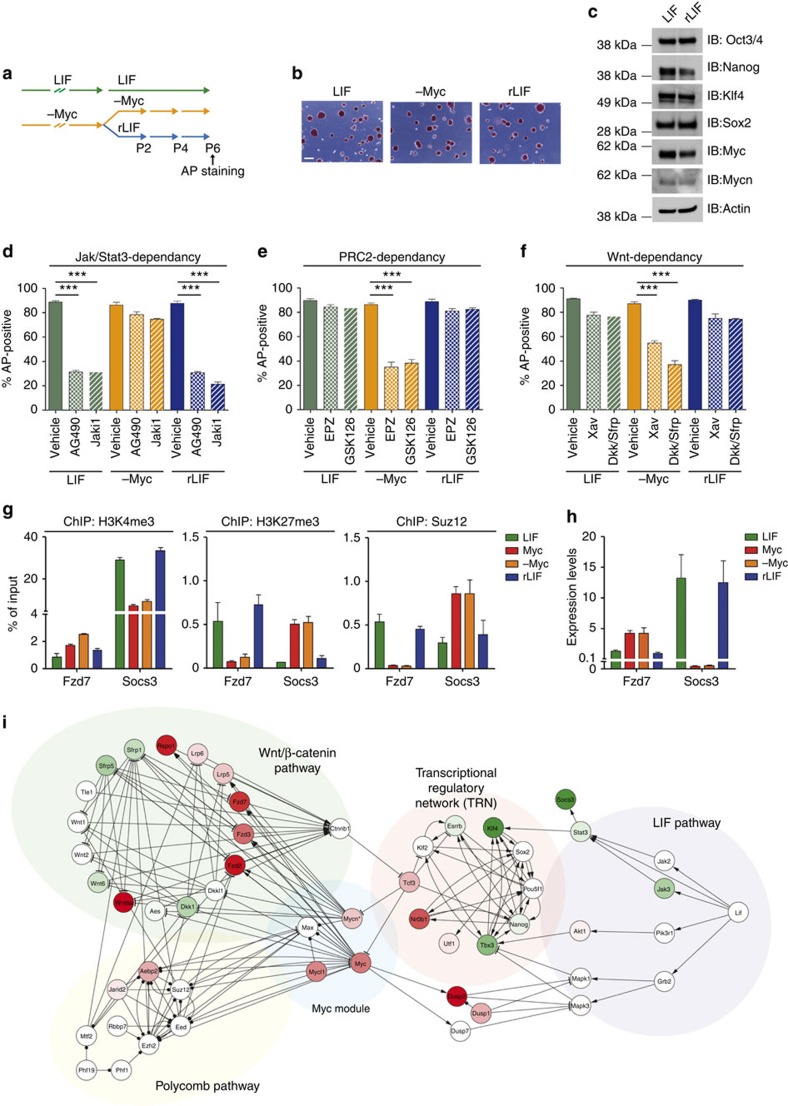
Myc-derived ESCs can be reverted to a LIF-dependent state. (**a**) Schematic representation of the experiment. R1 MycER cells were grown either in the presence of LIF (LIF, green line) or in the absence of both LIF and OHT, after OHT withdrawal (-Myc, orange line). These Myc-derived ESCs were either maintained in the same culture condition (-Myc) or they were reverted back to a LIF-containing culture medium (rLIF, blue line). Alkaline phosphatase (AP) staining was analysed at indicated time points. (**b**) Representative images of P staining of ESCs grown in the indicated conditions (scale bar, 200 μm. (**c**) Western blot analysis of pluripotency and Myc(s) transcription factors in LIF- and rLIF-ESCs. Immunostaining (IB) analysis was performed using the indicated antibodies; β-actin was used as loading control. (**d–f**) AP staining to assess Jak/Stat3- (**d**), PRC2- (**e**) and Wnt-dependency (**f**) of LIF-, -Myc- and rLIF-ESCs. Cells were grown in the absence (vehicle) or presence of indicated drugs (AG490 and Jaki1, EPZ and GSK126, Xav and Dkk1/sFRP1 were used to respectively inhibit Jak/Stat3 pathway, PRC2 activity and WNT pathway). (**g**) ChIP on LIF-, Myc-, -Myc- and rLIF-ESCs. The levels of H3K4me3, H3K27me3 and Suz12 at the TSS of the indicated genes were measured. (**h**) Relative transcriptional levels of Fzd7 and Socs3 in ESCs maintained in LIF-, Myc-, -Myc- and rLIF-ESCs, as measured by qRT–PCR analysis. Data in panels **d**–**h** are means±s.e.m. (*n*=3). (***P<0.001; Student's *t*-test). (**i**) Network visualization of the Myc-mediated machinery to sustain ESCs self-renewal. The four main molecular modules are shown, from right to left: the LIF signal transduction module, the core transcriptional regulatory network, the Wnt/β-catenin pathway and the Polycomb module. Network edges were manually curated according to literature knowledge, laboratory findings and completed querying interaction repositories: they indicate activation (arrowheads), inhibitions (T-shaped heads) and physical interactions (circle-shaped head). Microarray expression values (LIF/Myc ratios) of genes or RT–qPCR expression values from validation experiments (*) are overlaid onto network depicting the LIF/Myc ratios (from green for higher LIF to red for higher Myc).

To ultimately verify that the transcriptional programme of Myc-derived ESCs was depending on epigenetic and not on genetic changes, we attempt to revert the Myc-derived ESCs back to a LIF-dependent state (Fig. 7a). We observed that after several passages in LIF-conditioned medium, the Myc-derived ESCs reverted towards a LIF-dependent state (rLIF). Reverted-LIF ESCs formed dome-shaped and AP-positive colonies, expressing similar levels of pluripotency and Myc(s) transcription factors with respect to LIF-maintained ESCs (Fig. 7a–c). Although their maintenance was strictly depending on the Jak/Stat3 pathway as its brief inactivation led to cell differentiation (Fig. 7d), they resulted insensitive to the inhibition of Wnt signalling and PRC2 enzymatic activity (Fig. 7e,f). The signalling switch from Wnt to LIF dependency was mirrored by epigenetic changes on those genes involved in these pathways. By ChIP assay, we found that the modulator of Jak/Stat3 pathway Socs3, which gained *de novo* H3K27me3 deposition in Myc-maintained and Myc-derived ESCs, reacquired an active chromatin state as shown by the increased level of H3K4me3 and the simultaneous decrease of Suz12 and H3K27me3 at its promoter (Fig. 7g). On the contrary, the Wnt receptor Fzd7, which was activated upon transition to Myc-dependence state, was epigenetically repressed in rLIF-ESCs, similar to LIF-maintained ESCs (Fig. 7g). Of note, the epigenetic switch determined the transcriptional reactivation and repression of these modulators of LIF and Wnt pathways, respectively (Fig. 7h). Altogether, these results indicate that a prolonged MycER stimulation establishes a reversible epigenetic memory in Myc-derived ESCs, in which Wnt antagonists are maintained repressed by endogenous Myc proteins through PRC2 recruitment. This ultimately leads to reinforcement of the autocrine Wnt signalling pathway, which sustains maintenance of Myc-derived ESCs.

## Discussion

Self-renewing pluripotent stem cells are poised to differentiate into any cell type in response to transient signalling cascades, which drive the activation of downstream effectors that establish distinct gene expression patterns that are then stably maintained. Indeed, stem cell self-renewal and developmental transitions are followed by dynamic changes in the chromatin state, which then ensure the maintenance of cell identity through multiple rounds of cell divisions. Albeit of its importance, the molecular mechanisms governing the transition from a temporary to a stable and heritable gene expression programme in the absence of the instructive signals, are largely undefined.

In this work, we report the central role of Myc in establishing an epigenetic memory in ESCs by sustaining the self-reinforcing TRN via the potentiation of the Wnt/β-catenin pathway and the inhibition of autocrine Fgf4/Erk pathway, thus recapitulating the ground state of ESCs^15^ (Fig. 7i). We show that the activation of MycER establishes a positive feedback loop by repressing the Wnt antagonists via PRC2 recruitment, thus sustaining Wnt signalling, which triggers the transcriptional activation of the endogenous *Myc* and *Mycn* genes. Furthermore, we demonstrate that the Myc-driven self-reinforcing circuit induces an epigenetic memory in ESCs, which in turns could be propagated in the absence of further inputs from either the ectopically expressed MycER protein or from exogenous LIF signalling.

Despite the proven role of Myc in the establishment and maintenance of pluripotency^23^,^24^,^25^,^26^, its mechanisms of action are still not fully defined. It has been proposed that Myc, by regulating a clearly distinct set of genes from those regulated by core pluripotency factors, participates in sustaining self-renewal by enhancing growth, cell cycle progression and metabolisms of ESCs^11^,^27^. Other studies proposed that Myc acts as a general transcription amplifier by stimulating transcription elongation of all active genes^35^,^36^. Although these activities may be important for enhancing cellular growth and proliferation in cells overexpressing Myc, they could not explain the crucial role of Myc in maintaining ES cell identity. In addition, previous observation suggested that Myc could contribute to pluripotency by directly repressing primitive endoderm differentiation of pluripotent stem cells by directly inhibiting the transcriptional activation of Gata6 (ref. 25). Our results show that Myc sustains self-renewal by maintaining repressed those genes encoding for Wnt antagonists in cooperation with the PRC2 complex. In fact, we unveiled a critical interplay between Myc activation and the autocrine Wnt/β-catenin signalling which drives the onset of a positive feedback loop through the transcriptional activation of the endogenous *Myc* and *Mycn* genes. This regulatory circuitry stabilizes the TRN by counteracting the transcriptional repression activity of Tcf3, possibly through its association with β-catenin (Fig. 7i).

We demonstrated by functional analyses that the establishment of this self-reinforcing circuit is dependent on the integrity and biochemical activity of the PRC2. Importantly, we showed that PRC2-dependent impairment of Myc-ESC self-renewal was restored upon reactivation of Wnt pathway. These data highlight the biological significance of the Myc-dependent recruitment of the PRC2 to Wnt antagonists in ESCs. We demonstrated using different approaches that Myc associates with PRC2 complex through direct interaction with the core proteins Eed and Aebp2. Interestingly, among the PcG proteins, Aebp2 is the only transcription factor, which has been shown to have some degree of specificity in DNA binding^47^. The close association of Myc with Aebp2 within the PRC2 complex invokes the possibility of a synergy in the DNA-binding recognition of the Myc–PRC2 complex thus favouring the selection for specific loci. In addition, considering that Myc-dependent recruitment of the proper cofactor to a specific target gene is crucial for the transcriptional outcome, it is likely that a combination of events including intracellular signalling that induce post-translational modifications and/or the chromatin context may play an important role in the selection of alternative complexes.

Nevertheless, although we cannot exclude other mechanisms governing the Myc/PRC2 interplay^39^,^48^, we propose that Myc activation drives an alternative epigenetic state in ESCs by cooperating with PRC2 and this functional association is instrumental for the establishment of an epigenetic memory in the Myc-derived ESCs. Thus, our findings suggest an additional role of PRC2 in sustaining self-renewal of Myc-dependent ESCs, which differ from its function in lineage priming that have been previously described^34^,^49^. The apparent discrepancy between our findings and previous reports could be explained by considering that the role of PRC2 in self-renewal have been evaluated in ESCs grown in LIF-dependency^34^,^50^ or in 2i medium^51^ where other signalling cascades counterbalance the improper inhibition of the autocrine Wnt/β-catenin pathway as consequence of PRC2 inactivation. The finding that β-catenin^−/−^ ESCs could not self-renew in the absence of LIF stimulation^16^,^18^ support the notion that the balance between self-renew and differentiation potential is driven by different extrinsic and intrinsic inputs that thereby converge on chromatin to reinforce or weaken the self-propagating TRN. Our data indicate that alternative regulatory circuits can be established in response to the activation of the Wnt/β-catenin pathway and the concomitant inhibition of Fgf4/Erk signalling, as highlighted by the different epigenetic landscape of 2i-grown ESCs^52^. Distinct pluripotent stem cells can be accomplished depending on derivation and maintenance conditions, resulting in different epigenetic programmes that are activated and maintained in response to extrinsic and intrinsic signals. We here demonstrate that exogenous Myc induces epigenetic changes, which support an alternative pluripotent state whose maintenance depends on the reinforcement of the Wnt pathway. However, we do not exclude that other molecular mechanisms could be involved in inducing or stabilizing the Myc-derived ESC epigenetic state, thereby incrementing their self-renewal capacity and developmental potency. Finally, our data may resolve the previous conflicting observations showing that although Myc(s) loss is not compatible with the maintenance of stemness in cells grown in LIF dependency^25^,^26^, the Myc/Max complex is dispensable for preserving ESCs when exposed to the 2i medium 15,40,52.

In summary, our data explained the molecular mechanism through which a transient self-renewing signal, namely the activation of MycER in ESCs, could be converted into a long-lived epigenetic change by activating a Myc-driven positive feed-back loop, thus establishing an epigenetic memory in ESCs.

## Methods

### Cell culture conditions

R1 ESCs (obtained from American Type Culture Collection) were cultured without feeders on plastic coated with 0.1% gelatin in DMEM supplemented with 15% FCS (Hyclone Millipore, cat. ES-009-B), 100 mM 2-mercaptoethanol (Sigma, cat. M7522), 1 × MEM non-essential amino acids (Invitrogen, cat. 1140-036), 2 mM L-glutamine, 1 mM sodium pyruvate (Invitrogen), 100 μg ml^−1^ Vitamin C (L-ascorbic acid 2-phosphatase Sigma, A8960) and 100 U ml^−1^ LIF (Millipore, cat. ESG1107). The R1 Myc^T58A^ER ESCs (clone D1) were a generous gift from Dr Stephen Dalton and were maintained in ESC medium as described. The R1 MycER ESCs clone A2 was obtained upon transfection with pBABE-MycER vector followed by puromycin selection. The double-knock down for c- and N-Myc was obtained by lentivirus transduction followed by puromycin selection of MycER ESCs that were maintained in the presence of 50 nM OHT (Sigma, cat. H6278) and LIF. The knock-down for Eed, Ezh2 and β-catenin were obtained by lentivirus transduction followed by puromycin selection of MycER ESC.

GOF18 EpiSCs were derived from E5.5 epiblasts obtained from GOF18-eGFP mice and cultured in N2B27 medium supplemented with BSA, 0.033% Glutamax, 0.1 mM β-Mercaptoethanol, 12 ng ml^−1^ bFGF and 20 ng ml^−1^ Activin-A. EpiSC grown on fibronectin-coated plates and splitted every 3 days 1:20 by dissociation into small clumps with Collagenase Type IV 0.5 mg ml^−1^ (Gibco, cat. 17104-019).

ES colony-forming assays were carried out by plating 5000 ES cells per cm^2^ on 0.1% gelatin-coated plates and by growing cells for 3 days. After cell fixations, plates were stained for AP (Vector Lab., cat. SK-5100) according to the manufacturer's protocol and scanned with Nikon Eclipse T*i* instrument to score positive colonies. Relative quantification of positive colonies are always represented as percentage of the total colonies formed. Single-cell colony assay was performed by sorting viable cells into 96-well plates and plating one cell per well. Colonies were scored on the basis of morphology and AP staining after 1 week of cell culture in ESC medium.

EpiLCs were induced as previously described^29^. Briefly, ESCs maintained in 2i medium (N2B27 medium supplemented with CHIR99021 3 μM, and PD0325901 1 μM, Sigma) were plated on fibronectin-coated plates (16.7 mg ml^−1^) and grown for 3 days in N2B27 medium containing Activin A (20 ng ml^−1^), bFGF (12 ng ml^−1^) and knockout serum replacement (KSR) (1%).

All cell lines were tested by quantitative PCR to exclude mycoplasma contamination.

### Reagents

ESCs were treated with the following reagents in the described experimental procedures: 100 ng ml^−1^ Dkk1 (R&D, cat. 5897-DK); 100 ng ml^−1^ Sfrp1 (R&D, cat. 1384-SF); 100 ng ml^−1^ Wnt-3a (R&D, cat. 5036-WN); 12 ng ml^−1^ bFGF (Invitrogen, cat. 13256029); 20 ng ml^−1^ Activin-A (R&D, cat. 338-AC); 3 μM CHIR99021 (AbCam, cat. ab120890); 1 μM PD 0325901 (Sigma, cat. N°PZ0162); 1 μM EPZ005687 (BioVision, cat. 2364); 2 μM GSK126 (BioVision, cat. 2282); 5 μM AG490 (Invivogen, cat. N°tlrl-ag4); 1 μM Jak1 (Millipore, cat. N°420099); 3 μM XAV939 (AbCam, cat. N°ab120897).

### RNA extraction and analysis

Total RNAs were isolated from biological triplicates of ES cell cultures after 3 days growth in indicated conditions, using TRIzol Reagent (Ambion, cat.15596-026). Reverse transcription and PCR amplification were performed with the Superscript III Platinum One-Step qRT–PCR kit (Invitrogen, 11732-088) with the SYTO9 green fluorescent DNA dye (Invitrogen, S-34854). Quantitative reverse transcription–PCR (qRT–PCR) reactions were performed on a Rotor-Gene Q thermocycler (Qiagen, 9001560) and relative gene expression levels were determined using calculated concentration values, normalized to ERCC Spike-In Control RNA (Ambion, 4456740). For microarray experiments, 500 ng of each sample of RNA were processed to generate labelled cRNAs following the Illumina TotalPrep RNA amplification Kit (Ambion, AMIL1791) protocol. cRNA concentration was quantified and subjected to quality control on Agilent Bioanalyzer (Agilent Technologies, G2943CA) and hybridized to MouseRef-8 v2 BeadChip Arrays (Illumina, 1128893).

### Proximity ligation assay

ESCs were fixed in 4% paraformaldehyde (PFA) for 20 min, washed, blocked and incubated with primary antibodies from different species following the protocol procedure. PLA assay (Olink Bioscience) was performed by incubating secondary antibodies conjugated with oligonucleotides (probes) for 1 h at 37 °C followed by hybridization and ligation with the PLA probes. Amplification of the occurred ligation was performed by *in situ* amplification in the presence of fluorescent-labelled oligonucleotides. Images were acquired using Leica TCS SP5 confocal microscope, with HCX PL APO × 40/1.25 objective. The signal intensity of the proximity reactions was quantified using Volocity Analysis software (PerkinElmer).

### Protein extraction, western blots, protein immunoprecipitation and protein interactions

Total protein extracts were obtained as follows. Cells were washed twice with cold PBS, harvested by scrapping in 1 ml cold PBS and centrifuged for 5 min at 1,500 r.p.m. Harvested cell pellets were lysed by the addition of 5X (v/v) ice-cold F-buffer for 30 min at 4 °C. The chromosomal binding proteins were then separated using BioRuptor waterbath sonicator (Diagenode) at low setting for 5 min. Samples were sonicated in pulse of 30 s with 30 s intervals. Lysates were cleared by centrifugation for 10 min at 14,000 r.p.m. at 4 °C and supernatant was collected on ice. Protein concentration of lysates was determined using PierceTM BCA Protein Assay Kit (Thermo Scientific, cat. N°23227), according to the manufacturer's instructions. The absorbance was measured at *λ*=595 using SAFAS spectrophotometer (SAFAS, Monaco). Values were compared with a standard curve obtained from the BSA dilution series.

For western blot analysis, 20 μg of protein samples were boiled and loaded onto a pre-cast Bolt 4–12% Bis-Tris Plus gels (Novex, cat. N°NW04122BOX) and run in Bolt MES running buffer (Novex, cat. N°B0002). After electrophoresis, proteins were transferred to a nitrocellulose membrane. Membranes were blocked in PBS-T containing 5% Blotting-Grade Blocker (Bio-Rad, cat. N°170-6404; blocking buffer), for 1 h at room temperature (RT) with constant agitation and incubated with indicated primary antibody O/N at 4 °C with agitation. The membrane was then washed three times with PBS-T, each time for 5 min, followed by incubation with secondary antibody horseradish peroxidase-conjugated for 1 h at RT. ECL reagents (GE Healthcare, cat. N°RPN2232) was used to initiate the chemiluminescence of horseradish peroxidase. The chemiluminescent signal was captured using LAS3000 system (GE Healthcare). All uncropped western blots can be found in Supplementary Fig. 11.

Nuclear proteins were extracted by incubating isolated nuclei with F-buffer (10 mM Tris-HCl at pH 7.0, 100 mM NaCl, 30 mM Na Pyrophosphate, 50 mM NaF, 5 mM ZnCl_2_, 1% Triton X-100)^53^, briefly sonicated and treated with 10 U ml^−1^ DNaseI (Sigma) at 4 °C. Protein extracts were incubated with the specific antibody and the immunocomplexes were washed four times with F-buffer and twice with 0.15 M NaCl F-Buffer. Interacting proteins were eluted by incubating with 0.4 M NaCl TE buffer. For Flag-based IP, nuclear extracts were incubated with M2 Flag agarose resin (Sigma) overnight. Beads were washed four times with F-buffer and twice with 0.15 M NaCl F-Buffer and proteins were eluted by incubating with 0.5 mg ml^−1^ of 3xFlag peptide. Size exclusion chromatography was performed on a Superose-6 10/300 GL gel filtration column (GE Healthcare) in F-buffer using an AKTA purifier system (GE Healthcare).

### ChIP assay

Each ChIP experiment was performed in at least three independent biological samples as previously described^53^, with minor modifications. Briefly, cells were crosslinked with 1% formaldehyde for 10 min at RT and the reaction was quenched by glycine at a final concentration of 0.125 M, for 5 min at RT. Cells were lysed in lysis buffer (50 mM Tris-HCl pH 8, 0.1% SDS, 10 mM EDTA pH 8, 1 mM phenylmethyl sulphonyl fluoride (PMSF), protease inhibitor cocktail) and chromatin was sonicated to an average size of 0.1–0.5 kb, using a Branson D250 sonifier (4 cycles of 30 s, 20% amplitude). 50 μg of each sonicated chromatin was incubated O/N at 4 °C with 4 μg of indicated antibodies, reported in the Supplementary Information. Protein G-coupled Dynabeads were blocked O/N at 4 °C with 1 mg ml^−1^ sonicated salmon sperm DNA and 1 mg ml^−1^ BSA. Subsequently, blocked protein G-coupled Dynabeads were added to the ChIP reactions and incubated for 4 h at 4 °C. Dynabeads linked to ChIP reactions were then recovered and resuspended in RIPA buffer (10 mM Tris-HCl, pH 8, 0.1% SDS, 1 mM EDTA, pH 8, 140 mM NaCl, 1% sodium deoxycholate (DOC), 1% Triton, 1 mM PMSF, protease inhibitor cocktail). Magnetic beads were sequentially washed five times with ice-cold RIPA buffer, twice with ice-cold RIPA-500 buffer (10 mM Tris-HCl, pH 8, 0.1% SDS, 1 mM EDTA, pH 8, 500 mM NaCl, 1% DOC, 1% Triton, 1 mM PMSF, protease inhibitor cocktail), twice with ice-cold LiCl buffer (10 mM Tris-HCl, pH 8, 0.1% SDS, 1 mM EDTA, pH 8, 250 mM LiCl, 0.5% DOC, 0.5% NP-40, 1 mM PMSF, protease inhibitor cocktail) and once with TE buffer (10 mM Tris-HCl, pH 8, 1 mM EDTA, pH 8, 1 mM PMSF, protease inhibitor cocktail). Crosslinking was then reversed in direct elution buffer (10 mM Tris-HCl, pH 8, 0.5% SDS, 5 mM EDTA, pH 8, 300 mM NaCl) at 65 °C O/N. Finally, DNA was purified using SPRI beads, washed twice in EtOH 70% and dissolved in 60 μl of Tris-HCl, pH 8.0. DNA was analysed by quantitative real-time PCR using SYBR GreenER kit (Invitrogen). All experimental values were shown as percentage of input. To take into account background signals, we subtracted the values obtained with a non-immune serum to the relative ChIP signals. Oligonucleotide sequences will be provided upon request.

For β-catenin ChIP experiments, cells were serially cross-linked with 2 mM Di-N-succinimidylglutarate (Sigma 80424) for 20 min and 1% (v/v) formaldehyde for 10 min at RT. Crosslinking was stopped with 0.125 M (v/v) glycine for 10 min at RT. 4 μg of anti-bcat (200 mg ml^−1^; Santa Cruz, sc-7199 H102) were used for each ChIP assay.

### Time-lapse video microscopy

Time-lapse video microscopy and single-cell tracking of ESCs expressing H2B-eGFP were carried out continuously for 48 h at 37 °C and 5% CO_2_ using the Eclipse T*i* fully automated system (Nikon). Images of fluorescent cells were acquired every 20 min with 20 × Plan Apo λ objective (Nikon) using a LED illumination system combined with a CMOS camera (Andor) for the detection. Single-cell tracking was performed using the TTT software^54^ and movies were assembled using Image J software.

### Microarray analysis

BeadChip Arrays were scanned with HiScan Array Scanner (Illumina) using the iScan Control Software (Illumina). Genes and probes transcript levels were obtained from Illumina Intensity Data (.idat) files, applying quantile normalization and background subtraction implemented by the GenomeStudio Gene Expression Module v1.0 Software (Illumina). All experiments in each condition reported were performed on triplicate biological samples, except for the Eed knocked down ES grown in LIF withdrawal and in the presence of OHT, for which a single replicate was analysed on the array. Cutoffs for up- and downregulation of gene expression were set to 1.5-fold change threshold in all the analyses performed.

### Computational analysis of gene expression data

Single-microarray replicates were subjected to dendograms cluster analysis performed with the GenomeStudio Gene Expression Module v1.0 Software (Illumina), using Euclidean distance matrix. The top 200 genes up- or downregulated in the comparison between LIF maintained ES and EpiSC were selected to generate a heat-map reporting a hierarchical clustering analysis of both genes and single-microarray replicates (complete linkage, Euclidean distance matrix), using the TM4 MeV v4.9 software. To perform PCA with publicly available expression profiles^29^, our data were processed in the following ways. The data were corrected for background using negative control probes based on a normal-exponential convolution and scaled using quantile normalization and log2 transformation with R limma package. Then, the data were batch-corrected by cell type with the published data using ComBat. Furthermore, probes with more than twofold change in expression level between biological replicates were excluded, and the second EpiLC replicate was eliminated from the analysis because it is isolated from all of the other samples in the three-principal-component space making it difficult to see the pattern depicted in Fig. 2a. PCA was performed based on the correlation using R.

Differentially expressed genes in the LIF versus Myc maintained ES comparison were checked for biological and functional enrichment using both IPA (Qiagen, www.qiagen.com/ingenuity) and the Gene Ontology-based online tool PANTHER Classification System. GSEA ( http://www.broad.mit.edu/gsea/) was performed on genesets retrieved from both public available databases (Gene Ontology and the Kyoto Encyclopedia of Genes and Genomes, KEGG) and papers, as indicated in the list reported in Supplementary Data 1. For GSEA, the expression data were normalized using R limma package as described above, and the rank of genes based on log2 ratio of classes was used to identify significant gene sets using the weighted Kolmogorov–Smirnov test, in which phenotypes were permuted 10,000 times to obtain stable analysis results. Furthermore, we performed the PluriTest assay^33^ to identify the difference between the LIF and Myc-maintained ESCs in pluripotency-related gene expression patterns. To apply the PluriTest to our Illumina MouseRef-8 v2 BeadChip array samples, we employed the classifier constructed by 1,062 Affymetrix Mouse Gene 1.0 ST array samples^31^. Probe identifiers of the Illumina mouse bead array were matched to those of the Illumina human HT 12 bead array using BioMart. We followed the methods used in ref. 3 for data normalization and the calculation of pluripotency and lineage scores.

Network construction: a minimum set of 50 key genes belonging to molecular modules under investigation (LIF signal transduction, core pluripotency transcription factors, Myc, Wnt, beta-catenin and polycomb pathways) were selected and used as input nodes to build an interaction network using Cytoscape v.3.1.0. Manually curated interaction were added in the network annotation according to laboratory knowledge and findings, then network were refined and checked according to literature using the AgilentLiteratureSearch plugin and querying interaction databases in the GeneMania framework. Once network's interactions were assessed, gene expression data (LIF/Myc average signal ratios from Illumina microarrays) were overlaid to nodes and a force-directed layout was imposed to the network. Highly clustered genes/nodes (modules) in the obtained layout were then distributed with a circle layout for easier visualization; modules were manually emphasized (shading and labels) using Adobe Illustrator.

### ChIP-seq library generation and data analysis

Five nanograms of immunoprecipitaded and purified DNA were used to generate ChIP-seq libraries as previously described^55^, with minor modifications. Briefly, end repair of DNA fragments was achieved by sequential 15 min incubations at 12 °C and 25 °C with T4 PNK (10 U μl^−1^), T4 POL (3 U μl^−1^) and 0.1 mM dNTPs. A-base addition was performed by incubating end-repaired DNA fragments with Klenow (3′→5′ exo, 5 U μl^−1^) and 167 μM dATP for 30 min at 30 °C. Adaptor ligation was achieved by using the NEB Quick ligation kit (M2200L) and perfoming an incubation of 15 min at 25 °C. Processed DNA fragments were finally amplified with a thermal cycler for 14 cycles, by using the Agilent PfuUltra II Fusion HS DNA Pol kit (600674). All DNA purification steps between the different enzymatic reactions were performed by using Agencourt AMPure XP SPRI beads (Beckman, A63882). The obtained libraries were subjected to quality control on Agilent Bioanalyzer (Agilent Technologies, G2943CA) before sequencing them with Illumina HiSeq2000. Sequenced reads were aligned to the mouse genome (GRCm38/mm10) by using Bowtie2 version 2.2.3 (ref. 56) with default parameters; unique reads mapped to a best-matching location with no more than two mismatches were used in the subsequent analyses. We used MACS version 1.4.2 (ref. 57) to find the regions of ChIP-seq enrichment over background, and all parameters were set to their defaults in the peak-calling analysis. HOMER software^58^ was utilized to find peaks that are differentially enriched between LIF-derived and Myc-derived ES cell samples with the thresholds of >2.0-fold-change and <0.001 *P*-value. UCSC mm10 annotation was added to all the MACS-defined peaks and differentially enriched peaks with HOMER. TDF files were created by using IGVTools version 2.3.26 (ref. 59) to count reads in each 25 bp window, and the read coverage is normalized by per million mapped reads (RPM) on IGV^59^. HOMER and Java Tree View^60^ were used to generate heat maps of ChIP-seq enrichment around annotated transcriptional start sites (TSSs), and HOMER and R package ggplot2 were used to create histograms of the enrichment around TSSs. In the heat maps and histograms, the read coverage is normalized by RPM.

### Recombinant protein purification and pull-down experiments

Recombinant His-MYC and His-MYCΔMBII proteins were purified as follow: bacterial pellet was resuspended in Loading Buffer (10 mM HEPES, pH 7.9, 6 M guanidine and 5 mM β-mercaptoethanol) incubated for 1 h on a rocker wheel at RT and centrifuged for 30 min 23,700*g* at 4 °C. The supernatant was loaded on His-Trap column (GE), washed with 5 column volumes of BC500-7U (20 mM Tris-HCl, pH 7.9, 7 M Urea, 10% glycerol, 0.5 M KCl, 5 mM β-mercaptoethanol, 5 mM imidazole) followed by 3 column volume washes with BC100-7U containing 30 mM imidazole, and eluted with BC100-7 U containing 300 mM imidazole. Fractions containing recombinant proteins were dialysed in successive steps against BC100-4 U, -2 U, -1 U and finally -0 U buffer, all containing 0.1% NP-40.

For the Histidine pull-down experiment, 1 μg of purified His-MYC or His-ΔMBII were first bound to magnetic beads (Invitrogen) conjugated with 1 μg of anti-Histidine antibody and then incubated with 1 μg of GST fusion proteins in GST-binding buffer (40 mM HEPES, pH 7.3, 200 mM KCl, 0.1% Nonidet P-40, 0.2% Tween-20, 5 mM β-mercaptoethanol, inibitor protease cocktail) for 2 h at 4 °C. After extensive washes in the same buffer, the interacting proteins were eluted by heating the samples at 95 °C and analysed by immunoblotting.

### DNA constructs

The Flag-MYC and Flag-MYCΔMBII constructs have been previously described^53^. Myc, Mycn, β-catenin, Eed and Ezh2 Dkk1 and Sfrp1 shRNA constructs were obtained from Open Biosystem (TRCN0000042515, TRCN0000020694, TRCN0000012690 TRCN0000095722 and TRCN0000039042, respectively). The inducible IPTG-driven shRNA for Myc and Mycn were custom-made from Sigma-Aldrich (MISSION 3xLacO inducible shRNA system). For pull-down assay, the cDNAs corresponding to the human open reading frame of AEBP2, EED, EZH2, RBBP4, RBBP7 and SUZ12 were cloned into the bacterial expression vector pGEX-4T. To obtain the expression of the full-length EZH2 protein, the 3X FLAG tag was subcloned at the 3′ end of EZH2 cDNA. For FRET assay, EED and AEBP2 were cloned in pECFP-N1 or pEYFP-N1 (Clontech) vectors, positioning the fluorescence tags at C-termini, whereas NLS-, EZH2, MYC and MYCΔMBII were cloned into YFP-containing pcDNA5/FRT (Invitrogen) vector positioning the fluorescence tags at N-termini.

### Flow cytometry analysis (FACS)

Intracellular FACS quantification of Oct4 and Nanog protein levels was performed as previously described^12^ with minor modifications. Briefly, cells were dissociated with 0.25% trypsin and fixed first in 0.25% PFA in ES medium then in 70% methanol in PBS for 1 h at 4 °C. Cells were permeabilized and blocked in PBS/BSA 1%/Triton X-100 0.1%/goat serum10% (GIBCO Life Technologies, cat. no 16210-064) for 30 min at RT. The staining was performed overnight at 4 °C in the same buffer containing the indicated antibodies: anti-Oct4 (Santa Cruz, cat. no sc-5279) and anti-Nanog (Bethyl, cat. no A300-397A) to a final concentration of 2.5 and 10 μg ml^−1^, respectively. For the detection anti-mouse IgG-Alexa Fluor-647 (Invitrogen, cat. no A21236) and anti-rabbit IgG-Alexa Fluor-405 (Invitrogen, cat. no. A31556) were used for Oct4 and Nanog, respectively (1/200 for 2 h at RT).

For cell cycle analysis, trypsinized cells were washed with PBS, and cell nuclei DNA were stained with propidium iodide overnight using the DNA con 3 kit (Consul T.S.) and samples were analysed using the FACS Canto II (Becton&Dickinson).

### Immunostaining

Immunofluorescence experiments were performed on 4% PFA-fixed cells grown on fibronectin-coated coverslips using standard procedures. Images were acquired using a Leica TCS SP5 confocal microscope with HCX PL APO × 40/1.25 objective. The presented images are the maximal projections of the five central stacks of the total *z*-axis range acquired (between 12 and 18 μm, each stack was 0.5 μm size).

### Fluorescence resonance energy transfer (FRET)

NIH3T3 cells were transfected with the appropriate ratio between the fluorescence-tagged proteins to optimize protein distribution. MYC-interaction experiments were performed by co-expressing the MAX protein (MYC: MAX ratio 4:1) and the protein of interest. FRET acceptor photobleaching was carried out using the Leica TCS SP2 confocal microscope and apFRET software (Leica) according to the manufacturer's instructions. Fixed cells were analysed with a HCX PL APO lbd.BL 63.0 × 1.40 oil objective and × 8 zoom. The argon laser was tuned to 458 nm to excite CFP (PMT window 465–495 nm) and to 514 nm to excite YFP (PMT window 555–630 nm). The region of interest was bleached until intensity of 20% in the YFP channel using the 514 argon laser line.

### Teratoma assay

Teratoma formation was assessed by injecting intracranial ESCs (Lif, Myc, - Myc) in 7-week-old immuno-compromised female mice (CD1 nude). Briefly, 2 × 10^5^ cells suspended in 2 μl of DMEM were delivered into the right striatum by stereotactic injection. The following coordinates were used: antero-posterior=0; mediolateral=2.5 mm; dorso-ventral=3 mm. Mice were killed at different times from 2 to 4 weeks post injection, according to the cell line originally injected. Haematoxylin and eosin staining was performed on 5-μm-thick paraffin sections.

### Generation and analysis of chimeric mice

We generated Myc-derived ESCs and labelled them with H2B-mCherry recombinant protein. Myc-derived labelled ESCs were injected into morula stage of C57BL/6N embryos and reimplanted into 8-week-old pseudopregnant C57BL/6N female mice (Charles River Italia). Whole embryos at E10.5 and E15.5 were recovered and analysed for the mCherry signal by fluorescence microscopy. We evaluate the efficiency of Myc-derived ESCs to contribute to chimaerism by calculating the percentage of mCherry-labelled embryos out of all embryo obtained from injected morula (8/15).

### Karyotype assay

To perform the karyotype assay, ESCs at exponential growth phase were cultured in the presence of colcemid (0.1 mg ml^−1^) for 8 h. Cells were rinsed with PBS, trypsinized for 5 min and pelleted by centrifugation. Pellets were resuspended in 500 μl of hypotonic solution (0.56% KCl) dropwise and incubated at RT for 15 min. We then collected the cells by 2 min spinning at 2,000 r.p.m. and resuspended the pellet gently in cold methanol/acetic acid (3:1) fixative. Fixed ESC suspension was dropped onto microscope slide and chromosome spreads were visualized with 4,6-diamidino-2-phenylindole.

### Antibodies

The antibodies used in this work are purchased by Santa Cruz Biotechnology (anti-MYC sc-764 for immunoprecipitation assay, ChIP experiments and immunoblotting; anti-N-MYC sc-56729; anti-MAX sc-197 and sc-765; anti-E-Cadherin sc-7870; anti-Oct3/4 sc-5279; anti-GST sc-138; anti-His sc-8036; anti-Tle3 sc-9124; anti-Tle4 sc-365406; anti-Tcf-3 sc-8635; anti-ERα sc-544), by Millipore (anti-histone H3 06-755; anti-trimethyl histone H3 Lys4 07-473; anti-trimethyl histone H3 Lys27 07-449; anti-Eed 05-1320; anti-Ezh2 07-689), by Cell Signalling (anti-Suz12 D39F6; anti-Ezh2 AC22; Phospho-GSK-3β (Ser9) 9323; anti-GSK-3β 9315; anti Lrp6 3395; anti-Ph-Lrp6 2568; anti-Phospho-Akt (Ser473) 4060; anti-Phospho-Akt (Thr308) 2965; anti-Akt 2920; anti-Erk1/2 9102; anti-Ph-Erk1/2 4370; anti-Active β-catenin, 8814; anti-Axin1 2087; anti-Tcf1 2203) by Abcam (anti-Myc ab11917 for MycER immunoblotting; anti-Suz12 ab12073; anti-Aebp2 ab101655; anti-Utf1 ab24273) and by Bethyl laboratories (anti-Menin A300-105A; anti Ash2l A300-112A; anti-Rbbp5 A300-109A; anti-Nanog A300-397A), by BD Trasduction Laborotories (anti-β-catenin 610153); from Zymed anti-Lamin B1 33-2000; from R&D (anti-Sox2 AF2018; anti-Klf4 3158) and anti-Ring1B was provided by Dr Haruhiko Koseki (RIKEN Yokohama Institute, Japan).

### Ethical statement

Animals have been housed in the animal house at Hospital San Raffaele (HSR—Milan). The use of animals has followed the ethical guidelines by IACUC 681 approved by HSR internal committee and Italian Ministry of Health and according to the national laws.

### Statistical analysis

All the quantitative data are shown as means plus s.e.m. or s.d., as specified in each figure legend. No statistical method was used to predetermine sample size and all experiments were repeated at least three times with specific sample sizes reported in each figure legend. Data collection was conducted randomly. Statistical *P* values calculated by unpaired Student's *t*-test are indicated in figures and relative figure legends (where not differentially specified, *P<0.05, **P<0.01, ***P<0.001). *P* values <0.05 were considered significant. Data collection and analysis of all studies involving animals were conducted randomly but not blinding.

### Data availability

The Microarray and ChIP-seq data that support the findings of this study have been deposited in the NCBI Gene Expression Omnibus database under accession code GSE80558.

## Additional information

**How to cite this article:** Fagnocchi, L. *et al*. A Myc-driven self-reinforcing regulatory network maintains mouse embryonic stem cell identity. *Nat. Commun.* 7:11903 doi: 10.1038/ncomms11903 (2016).

## Supplementary Material

Supplementary Information Supplementary Figures 1-11

Supplementary Data 1 List of all genesets used in this study to perform computational analyses

Supplementary Movie 1 Continuous live imaging of ESCs expressing H2B-mCherry, grown in LIF dependency, at passage 1 (p1).

Supplementary Movie 2 Continuous live imaging of ES-MycER cells expressing H2B-mCherry, grown in Myc dependency (+OHT), at passage 1 (p1).

Supplementary Movie 3 Continuous live imaging of ESCs expressing H2B-mCherry, grown in LIF dependency, at passage 9 (p9).

Supplementary Movie 4 Continuous live imaging of ES-MycER cells expressing H2B-mCherry, grown in Myc dependency (+OHT), at passage 9 (p9).

Supplementary Movie 5 Continuous live imaging of Myc-derived ESCs expressing H2B-mCherry, grown in absence of both LIF and/or OHT stimulation, at passage 9 (p9).
