# Supplementary material for: A Myc-driven self-reinforcing regulatory network maintains mouse embryonic stem cell identity
Source: Nat Commun. 2016 Jun 15;7:11903. doi: 10.1038/ncomms11903 (PMC4912626; doi:10.1038/ncomms11903)
Supplement: Supplementary Information — Supplementary Figures 1-11 [file ncomms11903-s7.pdf]

Fagnocchi_Supplementary Figure 1

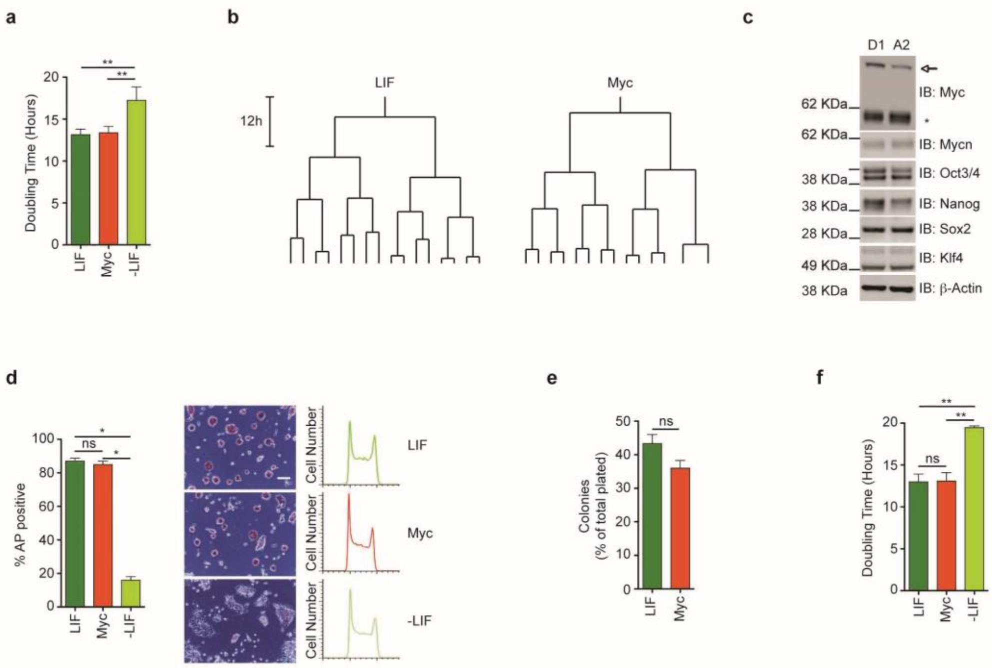

Supplementary Figure 1. Characterization of the independently derived MycER ESC A2 clone

(**a**) Quantification of the doubling time of MycER^{T58A} ESC D1 clone maintained in presence of LIF, OHT (Myc) or in absence of both LIF and OHT (-LIF). (**b**) Single-cell tracking of ESCs expressing H2B-eGFP grown in LIF and Myc-dependency for 48h. Lineage trees represent the pattern and timing of cell division of a single ESC grown in the indicated conditions. (**c**) Protein extracts were obtained from the R1 MycER^{T58A} ESCs clone D1 and from the newly derived R1 MycER ESCs clone A2, both maintained in presence of OHT. Immunoblot analysis was performed with the indicated antibodies to measure the relative protein levels. The arrow indicates the MycER fusion protein, while the asterisk indicates endogenous Myc. β-actin was used as loading control. (**d**) Alkaline Phosphatase (AP) staining of the MycER ESCs clone A2 grown for three days in the indicated conditions and relative quantification of positive colonies are represented as percentage of the total colonies formed. Representative images of stained ESCs maintained for three days in LIF, OHT (Myc) or in -LIF (middle panels) and the relative cell cycle profiles (right panels) are shown. Scale bar, 200 μm. (**e**) Single-cell clonogenic assay of MycER ESCs clone A2 was performed and the relative number of colonies grown in the indicated conditions was quantified and is represented as percentage of total number of single-cell plated cells grown. (**f**) Quantification of the doubling time MycER ESCs clone A2 maintained in presence of LIF, OHT or in absence of both LIF and OHT. Data in panels (**a**) and (**d-f**) are means +/- s.e.m. (n=3). (*P<0.05, **P<0.01; ns, not significant; Student's t test). Related to Figure 1.

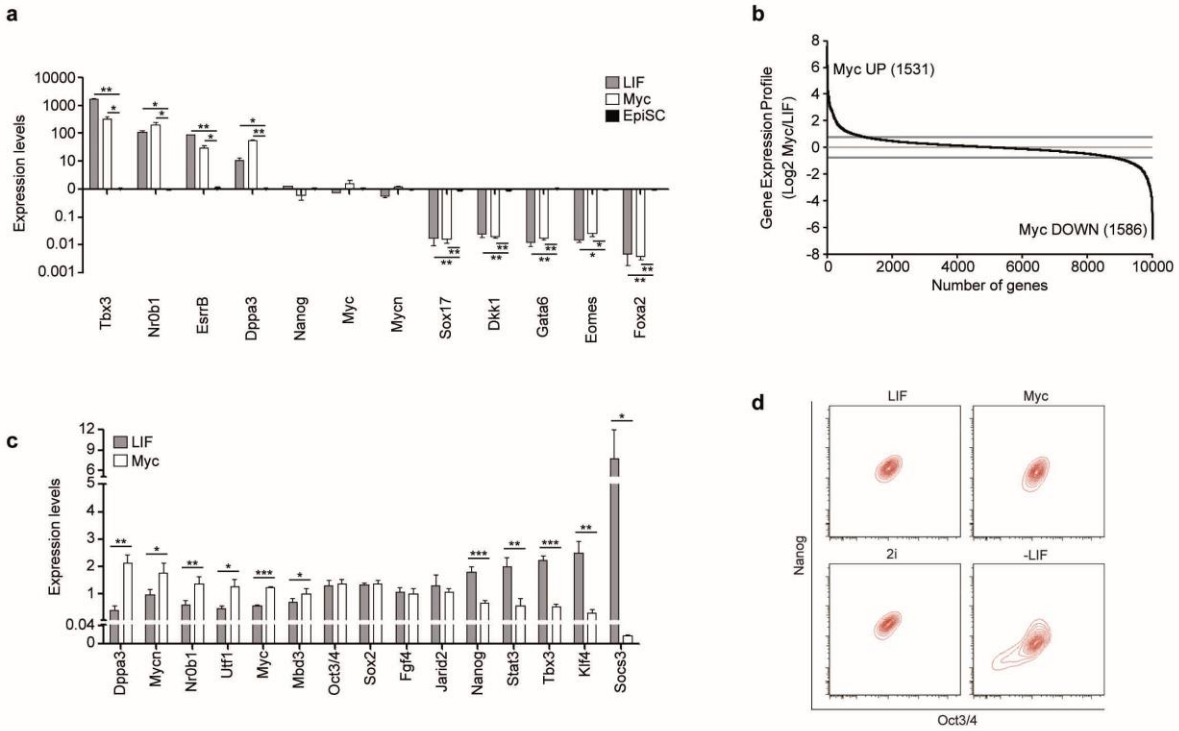

e

Myc up-regulated genes

GO Biological Processes

Term	P-value
Cellular process	1.3E-11
Metabolic process	1.3E-09
Primary metabolic process	1.1E-07
Cell cycle	1.7E-07
Nucleobase, nucleoside, nucleotide and nucleic acid metabolic process	1.8E-07
Developmental process	7.7E-07
Intracellular signaling cascade	1.1E-06
Regulation of transcription from RNA polymerase II promoter	1.3E-06
Transcription from RNA polymerase II promoter	5.8E-06
Transcription	1.1E-05
Signal transduction	7.4E-04

Myc down-regulated genes

GO Biological Processes

Term	P-value
Metabolic process	4.8E-10
Developmental process	1.8E-08
Primary metabolic process	2.0E-08
Cellular process	9.4E-07
System development	1.9E-06
Cell communication	3.1E-06
Signal transduction	1.8E-05
Transport	2.3E-05
Intracellular signaling cascade	2.9E-05
Ectoderm development	4.8E-05
Cell-cell signaling	4.4E-04
Mesoderm development	9.0E-04

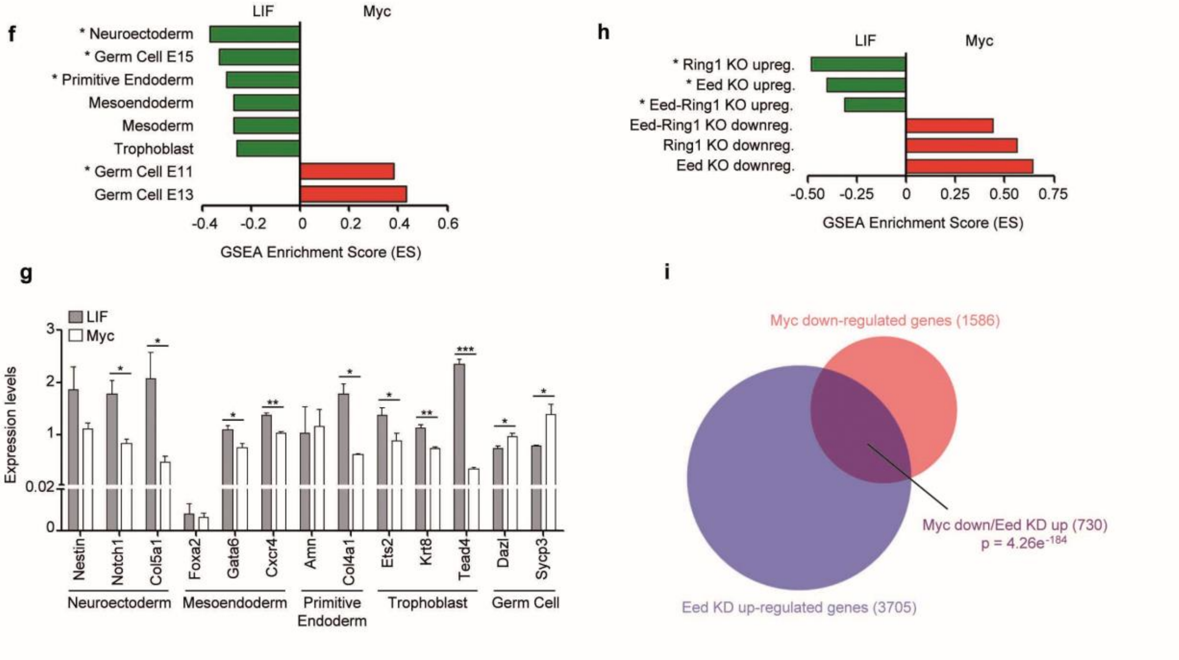

Fagnocchi_Supplementary Figure 2

Supplementary Figure 2. Gene expression profile of LIF- and Myc-ESCs

(**a**) Relative transcriptional levels of a set of ESCs- or EpiSC-specific genes were confirmed by qRT-PCR analysis in the indicated conditions. (**b**) Fold change (log_2 values) in gene expression level of all genes in Myc-versus LIF-maintained cells were obtained from triplicates and averaged. The relative ratio was calculated and the grey lines indicate the limits of fold change >1.5 and <-1.5. (**c**) Relative transcriptional levels of a set of pluripotency genes were confirmed by qRT-PCR analysis in the indicated conditions. (**d**) Intracellular FACS quantification of Oct3/4 and Nanog protein levels in MycER ESC grown in LIF, 2i, OHT (Myc) and in absence of both LIF and OHT (-LIF) conditions. (**e**) Functional annotation analysis of differentially regulated genes between LIF and Myc-maintained ESCs, showing Gene Ontology (GO) biological processes. (**f**) Representation of gene set enrichment analysis (GSEA) results using lineage-restricted gene sets in Myc- versus LIF-maintained ESCs (n=3; *P<0.0001). Genesets used are listed in Table S1. (**g**) The relative transcriptional levels of some developmental genes were confirmed by qRT-PCR analysis in the indicated conditions. (**h**) Representation of gene set enrichment analysis (GSEA) results using Polycomb (Eed and Ring1b) target gene sets in Myc- versus LIF-maintained ESCs (n=3; *P<0.0001). Genesets used are listed in Table S1. (**i**) Venn diagram representing the overlap between Myc down-regulated and Eed-repressed genes in ESCs. The p-value relative to the hypergeometric probability of Myc downregulated and Eed-repressed genes in Myc-ESCs is reported; total genes = 22592. Data in panels (**a**), (**c**) and (**g**) are means +/- s.e.m. (n=3). (*P<0.05, **P<0.01; ***P<0.001; Student's t test). Related to Figure 1.

a

LIF vs LIF+Myc
(Myc-induced)

IPA pathways

Term	P-value
Regulation of the Epithelial-Mesenchymal Transition Pathway	4.0E-05
Integrin Signaling	1.1E-04
EGF Signaling	7.7E-04
PI3K/AKT Signaling	1.5E-03
VEGF Signaling	2.5E-03
FGF Signaling	4.2E-03
Mouse Embryonic Stem Cell Pluripotency	4.2E-03
Human Embryonic Stem Cell Pluripotency	9.8E-03
Wnt/b-catenin Signaling	2.3E-02

GO Biological Processes

Term	P-value
metabolic process	8.94E-19
cellular process	1.19E-14
primary metabolic process	1.53E-14
cellular component organization	2.15E-08
cellular component organization or biogenesis	5.34E-08
biological regulation	3.19E-07
intracellular protein transport	1.26E-05
nucleobase-containing compound metabolic process	1.27E-05
protein transport	1.51E-05
cellular protein modification process	2.01E-05

b

Myc vs LIF+Myc
(LIF-induced)

IPA pathways

Term	P-value
STAT3 Pathway	3.9E-05
JAK/Stat Signaling	1.1E-04
Regulation of the Epithelial-Mesenchymal Transition Pathway	1.1E-03
Role of JAK1, JAK2 and TYK2 in Interferon Signaling	1.4E-03
PI3K/AKT Signaling	7.5E-03
Integrin Signaling	1.5E-02

GO Biological Processes

Term	P-value
metabolic process	1.9E-15
primary metabolic process	4.9E-14
cellular process	1.8E-12
cellular protein modification process	5.8E-06
biological regulation	1.1E-05
phosphate-containing compound metabolic process	1.6E-05
developmental process	3.8E-05
localization	7.4E-05
cellular component organization	1.7E-04
cell communication	2.7E-04

c

Oct3/4-Sox2-Nanog-Tcf3 Co-bound

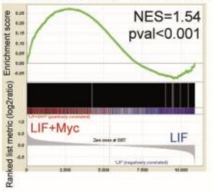

d

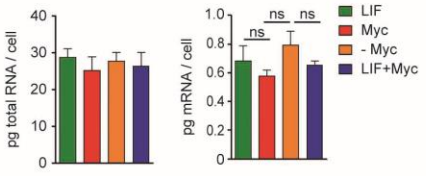

Fagnocchi_Supplementary Figure 3

Supplementary Figure 3. Validation of LIF- and Myc-specific pathways in the gene expression profile analysis

(**a**) Quantification of total and messenger RNA/cell in LIF-, Myc-, -Myc and LIF+Myc-maintained ESCs. Data are means +/- s.e.m. (n=3). (ns, not significant; Student's t test). (**b**) Ingenuity Pathway (IPA) and Gene Ontology (GO) analysss relative to Myc-induced genes, in the differential comparison between LIF- and LIF+Myc-maintained ESCs (n=3). (**c**) Ingenuity Pathway (IPA) and Gene Ontology (GO) analysis relative to LIF-induced genes, in the differential comparison between Myc- and LIF+Myc-maintained ESCs (n=3). (**d**) Gene set enrichment analysis (GSEA) of Oct4/Sox2/Nanog/Tcf3 common target genes in LIF+Myc-versus LIF-maintained ESCs (n=3). Related to Figure 1.

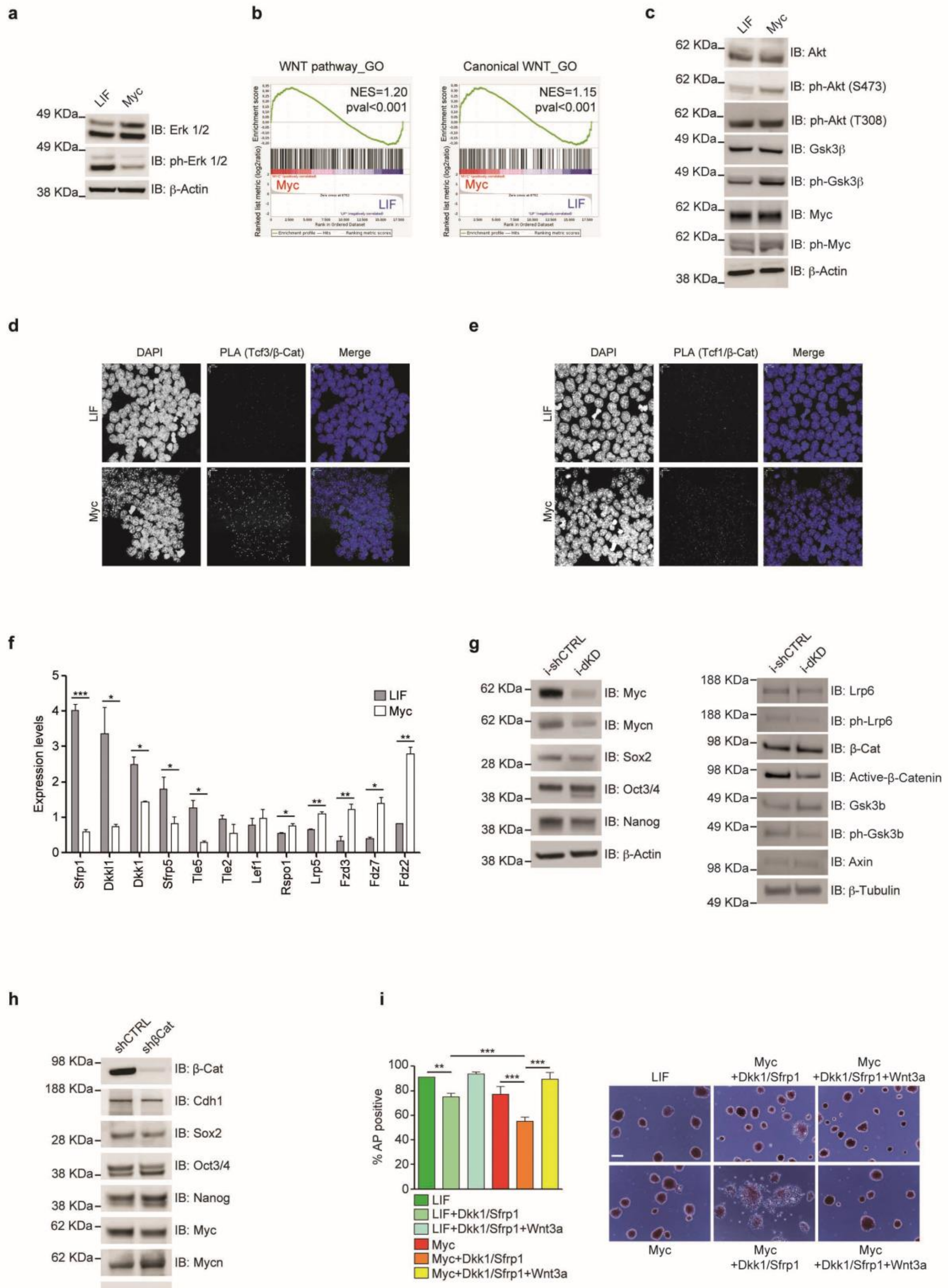

Fagnocchi_Supplementary Fig.4

Supplementary Figure 4. Myc sustains Wnt pathway activation

(**a**) Western Blot analysis of MAPK/Erk pathway related proteins. Total protein extracts were collected from LIF and Myc-maintained ESCs, and immunostaining (IB) analysis was performed using the indicated antibodies; β-actin was used as loading control. (**b**) Gene set enrichment analysis (GSEA) of Wnt pathway associated gene sets in Myc- versus LIF-maintained ESC (n=3). (**c**) Western Blot analysis of Akt/Gsk3β pathway related proteins in LIF and Myc-maintained ESCs by performing immunoblotting with the indicated antibodies; β-actin was used as loading control. (**d-e**) Representative images of *in situ* Proximity Ligation assay (PLA) between Tcf3 (**d**) or Tcf1 (**e**) and β-catenin within cell nuclei of LIF- and Myc-maintained ESCs; scale bar, 50 μm. (**f**) The relative transcriptional levels of a set of Wnt pathway associated genes were confirmed by qRT-PCR analysis in the indicated conditions. (**g**) Western Blot analysis of proteins extracted from MycER ESCs expressing either an IPTG-inducible control (shCtrl) or an IPTG-inducible double Myc and Mycn shRNAs (dKD). Immunostaining (IB) analysis was performed using the indicated antibodies; β-actin and β-tubulin were used as loading controls. (**h**) Western Blot analysis of proteins extracted from MycER ESCs expressing either a control or a β-catenin shRNA. Immunostaining (IB) analysis was performed using the indicated antibodies; β-actin was used as loading control. (**i**) Alkaline Phosphatase (AP) staining of R1 ES MycER cells grown for three days in presence or absence of Wnt pathway inhibitors Dkk1 and Sfrp1 or the Wnt3a ligand, as indicated. Relative quantification of positive colonies is represented as percentage of the total colonies formed. Representative images of stained ESCs are shown; scale bar, 200 μm. Data in panels (**f**) and (**i**) are means +/- s.e.m. (n=3). (*$P<0.05$, **$P<0.01$, ***$P<0.001$; Student's t test). Related to Figure 2.

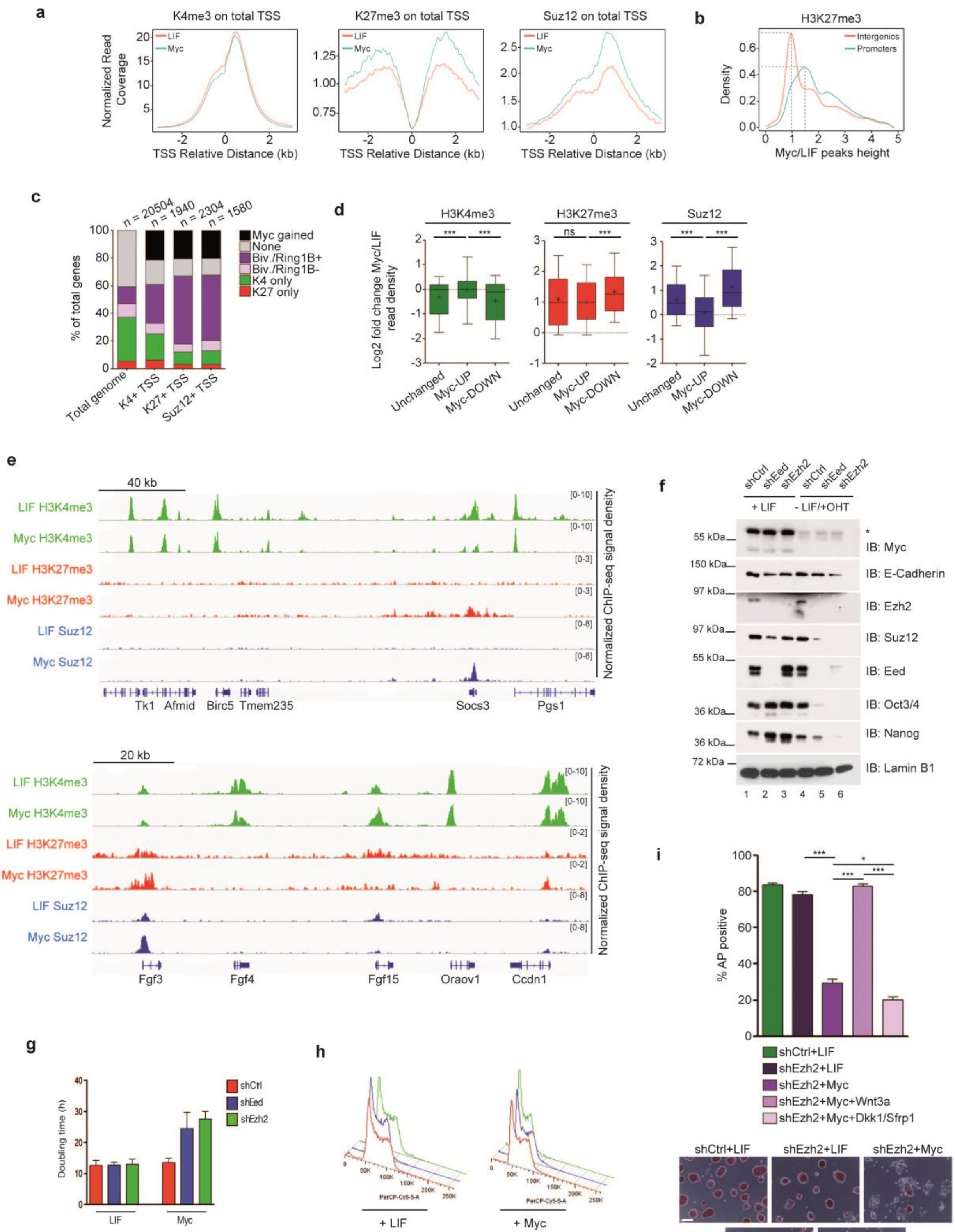

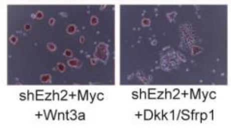

Fagnocchi_Supplementary Figure 5

Supplementary Figure 5. Genome wide Myc-driven modulation of the epigenetic state in ESCs

(**a**) Histograms showing the average profile of H3K4me3, H3K27me3 and Suz12 in LIF- and Myc-ESCs, centered on TSS of total genes. Window size is ±3 kb with 10 bp bin size. (**b**) Ratio of H3K27me3 peak heights in Myc/LIF ESCs at promoters and intergenic peaks. X-axis values >1 indicate augment of H3K27me3 in Myc-ESCs, compared to LIF-ESCs. Dashed lines indicate the most represented Myc/LIF peak height for intergenic and promoter regions. (**c**) Bar chart plot showing the chromatin state of genes which gained either H3K4me3,H3K27me3 or Suz12 in Myc-ESCs, compared to the reference distribution of epigenetic marks on the total genome. (**d**) Box-and-whisker plots of the Myc/LIF ChIP-seq read density fold-change on genes that are unchanged, or up- or down-regulated in Myc-ESCs. Boxes encompass the 25th to 75th percentile; whiskers extend to 10th and 90th percentiles. A central horizontal bar and a black cross indicate the median fold change and the mean, respectively. (ns = not significant, ***$P<0.001$; Student's t test). (**e**) Genomic snapshots showing H3K4me3, H3K27me3 and Suz12 binding in LIF- and Myc-ESCs. The x- and y-axis correspond to genomic location and ChIP-seq signal density normalized to sequencing depth, respectively. (**f**) Protein extracts were obtained from the MycER ESCs expressing either a control (Ctrl), Eed (shEed) or Ezh2 (shEzh2) shRNAs maintained in the presence of LIF or treated with OHT. Immunoblot analysis was performed with the indicated antibodies. * indicates the band at the expected molecular weight for endogenous Myc. (**g-h**) Quantification of the doubling time (**g**) and cell cycle profile (**h**) of MycER ES cell clones expressing either a control (shCtrl), or Eed (shEed) and Ezh2 (shEzh2) shRNAs maintained in the presence of LIF or treated with OHT. (**i**) AP staining of MycER ESC clones expressing either a control shRNA (shCtrl) or Ezh2 shRNAs (shEzh2) and grown in the indicated conditions. Representative images of stained ESCs grown in the

different conditions are shown. Scale bar, 200 μm. Data in panel (**i**) are means +/- s.e.m. (n=3). (*P<0.05, ***P<0.001; Student's t test). Related to Figure 3.

Fagnocchi_Supplementary Figure 6

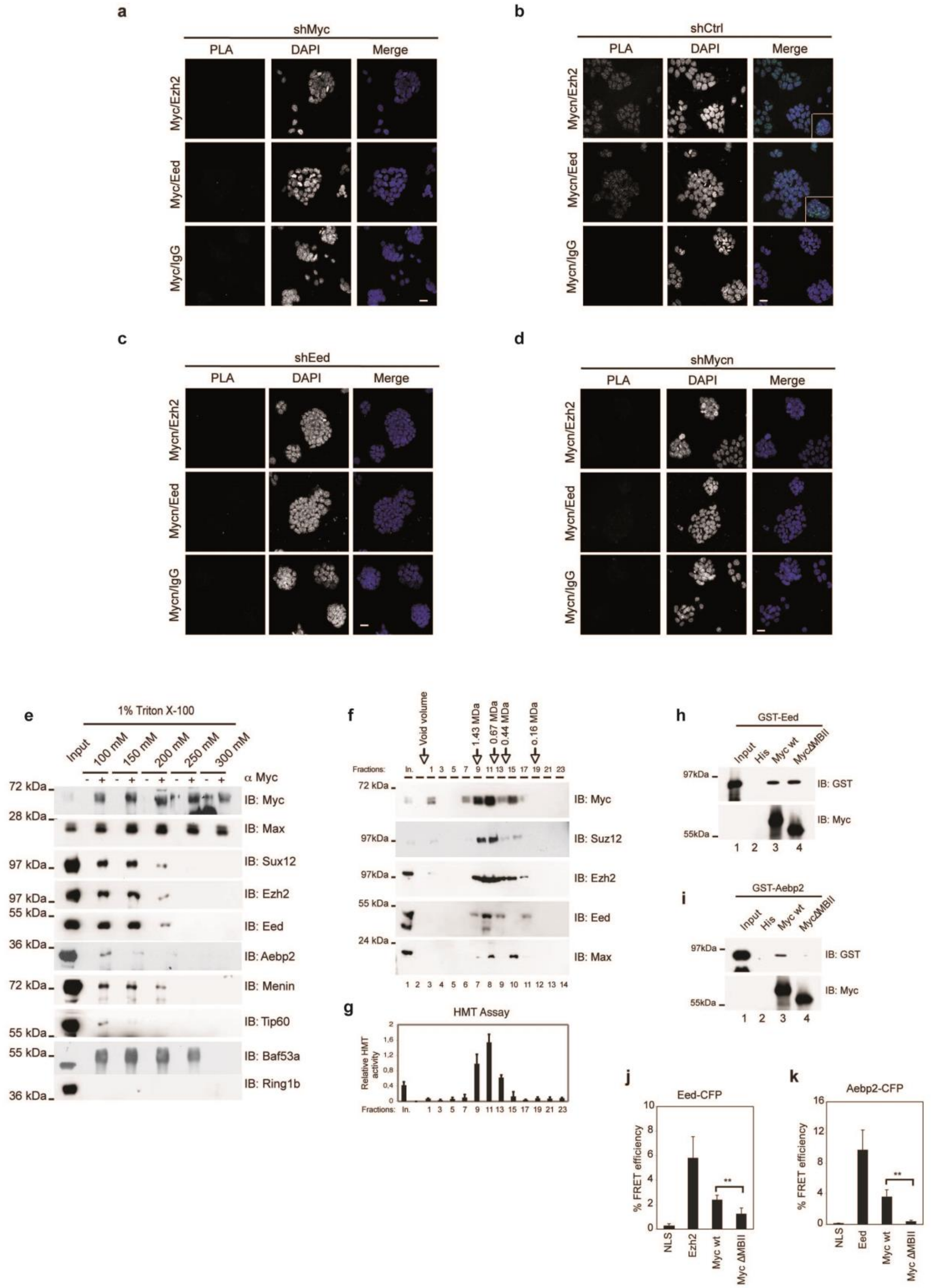

Supplementary Figure 6. Both Myc and Mycn interact with PRC2 in ESCs

(**a**) *In situ* Proximity Ligation assay (PLA) performed to measure the proximity between Ezh2 or Eed and Myc within cell nuclei of LIF-maintained ESCs, expressing a Myc (shMyc) shRNA. (**b-d**) *In situ* PLA was performed to measure the proximity between Ezh2 or Eed and Mycn within cell nuclei of LIF-maintained ESCs, expressing either a control shRNA (shCtrl, **b**), or Eed shRNAs (shEed, **c**), or a Mycn (shMycn, **d**). In all PLA, the proximity between Myc or Mycn and an unrelated protein (IgG) was assessed as negative control. Scale bar, 50 µm. (**e**) We measured the binding affinity between Myc and PRC2 components in ESCs by performing Co-IP experiments in presence of increasing amount of NaCl and 1% Triton X-100, as indicated. Nuclear extracts obtained from ESCs were either immunostained (Input) or subjected to immunoprecipitation (IP) with the anti-Myc antibody at different salt concentrations. Immunostaining (IB) analysis was performed using the indicated antibodies. 10% of the total protein samples were loaded as input. (**f**) Stable HEK 293 cell clones expressing the Flag-Myc construct were used to purify the Myc-interacting protein complexes after affinity chromatography purification. The Flag peptide-eluted protein complexes were separated by gel filtration and the obtained fractions were analyzed by immunostaining with the indicated antibodies. (**g**) H3K27-histone methyltransferase activities (HMT) of the obtained fraction were measured through a colorimetric histone methyltransferases assay. (**h-i**) The Eed and Aebp2 purified GST-fusion proteins were incubated with the His-Myc wt or His-MycΔMBII and the interacting proteins were revealed by immunostaining with the indicated antibodies. (**j-k**) The Fluorescence Resonance Energy Transfer (FRET) efficiency between Eed- or Aebp2-CFP and the indicated YFP-fusion proteins was calculated and the data are represented as the mean and the relative standard deviation obtained by analyzing 12 samples in three independent biological replicates. The student's T-test was applied to determine the probability (**= $p<0.001$). Related to Figure 4.

Fagnocchi_Supplementary Figure 7

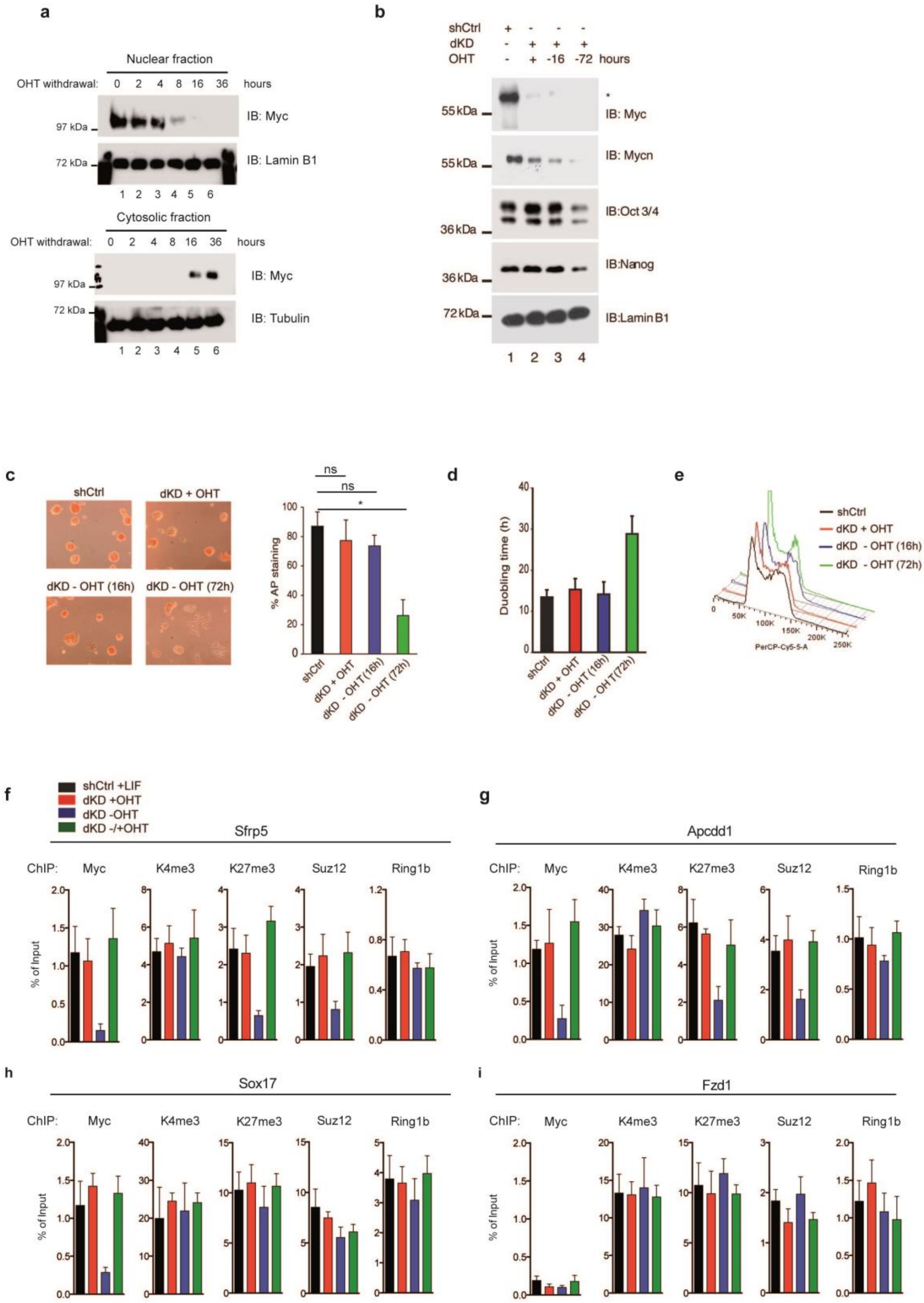

Supplementary Figure 7. Myc recruits PRC2 onto the chromatin

(**a**) Cytosolic and nuclear fractions were obtained from MycER ES cells clones expressing Myc and Mycn shRNAs (dKD) maintained in presence of OHT (0) or after OHT withdrawal at different time points, as indicated. Immunoblot analysis (IB) was performed with the indicated antibodies to measure the relative levels of MycER fusion protein and fractionation controls (Lamin B1 and Tubulin). (**b**) Protein extracts were obtained from the MycER ESC clones expressing either a control shRNA (shCtrl) or Myc and Mycn shRNAs (dKD) maintained either in the presence of OHT or after OHT withdrawal for 16 and 72 hours. Immunoblot analysis was performed with the indicated antibodies to measure the relative protein levels. * indicates the band at the expected molecular weight for endogenous Myc. (**c**) The alkaline phosphatase (AP) activity of the MycER ESC shCtrl and dKD clones was measured in the indicated culture conditions. (**d**) Quantification of the doubling time of shCtrl and dKD MycER ESC clones maintained either in presence of OHT or after OHT withdrawal for 16 and 72 hours. (**e**) Cell cycle profile of shCtrl and dKD MycER ESC clones cultured in indicated conditions. (**f-i**) Chromatin extracts were obtained from the MycER ESCs expressing either a control shRNA (Ctrl) or Myc and Mycn shRNAs (dKD). The level of K4me3, K27me3, and the binding of Myc, Suz12, Ring1b at the TSS of the indicated genes was measured by ChIP assay in the control cells (black bars), in the dKD cells maintained in the presence of OHT (red bars), or after OHT withdrawal for 16 hours (blue bars) followed by the re-induction of MycER by OHT treatment for additional 4 hours (green bars). Data in panels (**c**), (**d**) and (**f-i**) are means +/- s.d. (n=3). (*$P<0.05$, ns = not significant; Student's t test). Related to Figure 4.

Fagnocchi_Supplementary Figure 8

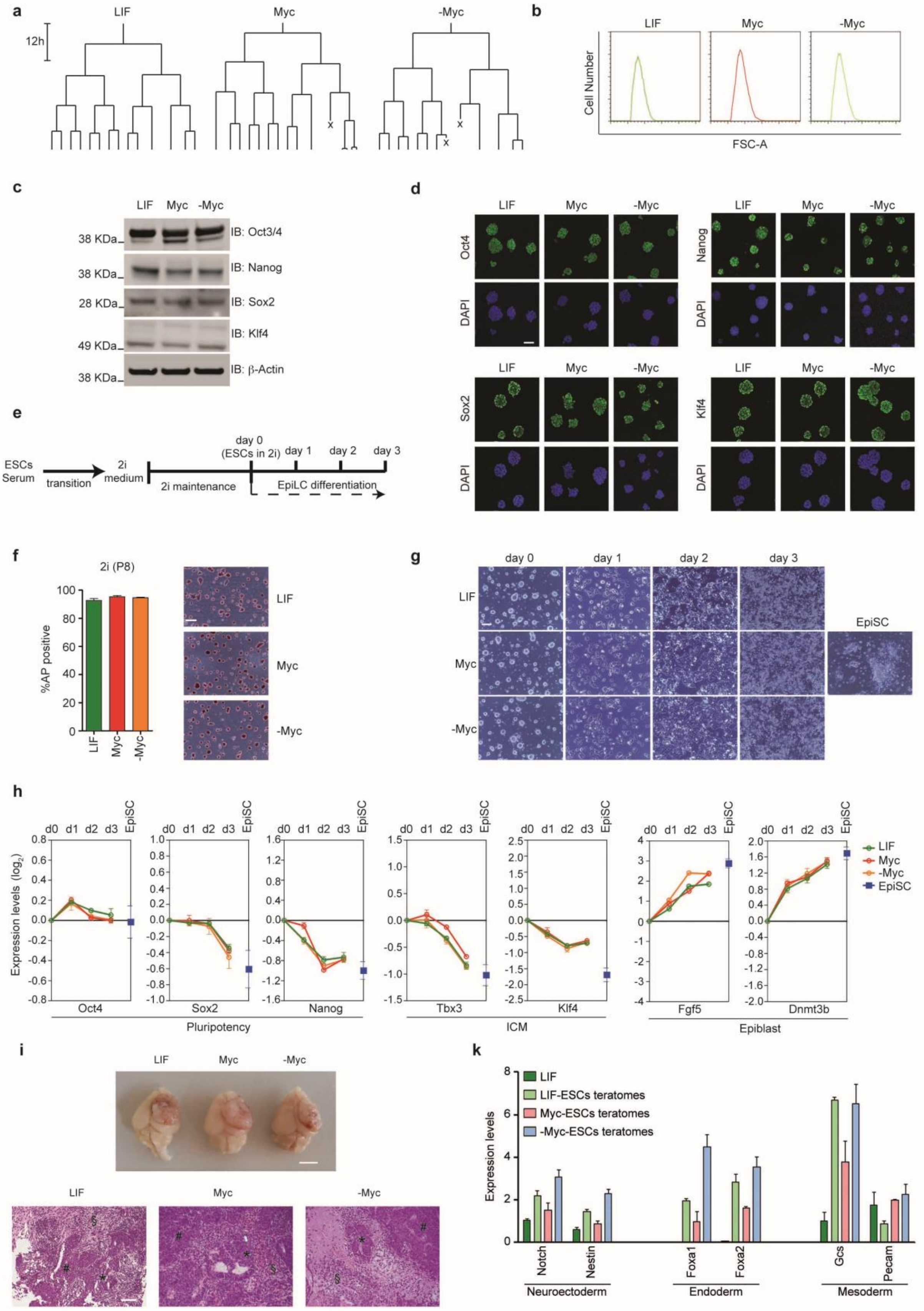

Supplementary Figure 8. Myc-derived ESCs retains self-renewal capacity, differentiation potential and pluripotency

(**a**) Single-cell tracking of LIF- or Myc-maintained (+Myc) and Myc-derived ESC (-Myc) expressing H2B-mCherry. Lineage tree represents the pattern and timing of cell division of a single ESC grown in the indicated conditions. (**b**) FACS analysis of cell size distribution (FSC) of ESC maintained for nine passages in LIF-, Myc-dependency or in Myc-derived ESC (-Myc). (**c**) Western Blot analysis of proteins extracted from ESC maintained in LIF-, Myc-dependency or in Myc-derived ESCs (-Myc). Immunostaining (IB) analysis was performed using the indicated antibodies; (**d**) Immunofluorescence analysis of Oct4, Nanog, Klf4 and Sox2 in LIF-, Myc-maintained and in Myc-derived ESC; scale bar, 50 μm. (**e**) Schematic representation of the epiblast-like cell (EpiLC) induction. (**f**) R1 Myc-ER cells maintained with LIF (LIF), OHT (Myc) or in absence of both LIF and OHT (-Myc) were grown in 2i for eight passages and alkaline phosphatase (AP) positive colonies were quantified. Data are means +/- s.d. (n=3). Scale bar, 200 μm. (**g**) Representative images taken at each day during EpiLC induction. Scale bar, 200 μm. (**h**) Quantification of the transcriptional levels of indicated genes in 2i-maintained ESCs (d0), during the EpiLC differentiation (d1-3) and in EpiSCs, The experiment was performed starting with LIF-, (green open circles), Myc- (red open circles) or Myc-derived ESCs (-Myc, orange open circles). Transcription levels in ESCs were set to 0 and all values are expressed in log2 scale. Data are means +/- s.d. (n=3). (**i**) Teratoma assay in Nude mice upon intracranial injection of LIF- (LIF), Myc-dependent (Myc) and Myc-derived (-Myc) ESCs (upper panel; scale bar, 5 mm). Haematoxylin/Eosin staining showed mesodermal- (mesenchymal-like; §), ectodermal- (epithelial-like; #) and endodermal-derived (gut-like; *) cell types in each Teratoma (lower panel; scale bar, 200 μm). (**k**) The relative transcriptional levels of a set of lineage-restricted genes were quantified by qRT-PCR analysis in the formed

teratomas and in LIF-ESCs, as a comparison. Data are means +/- s.e.m. (n=3). Related to Figure 6.

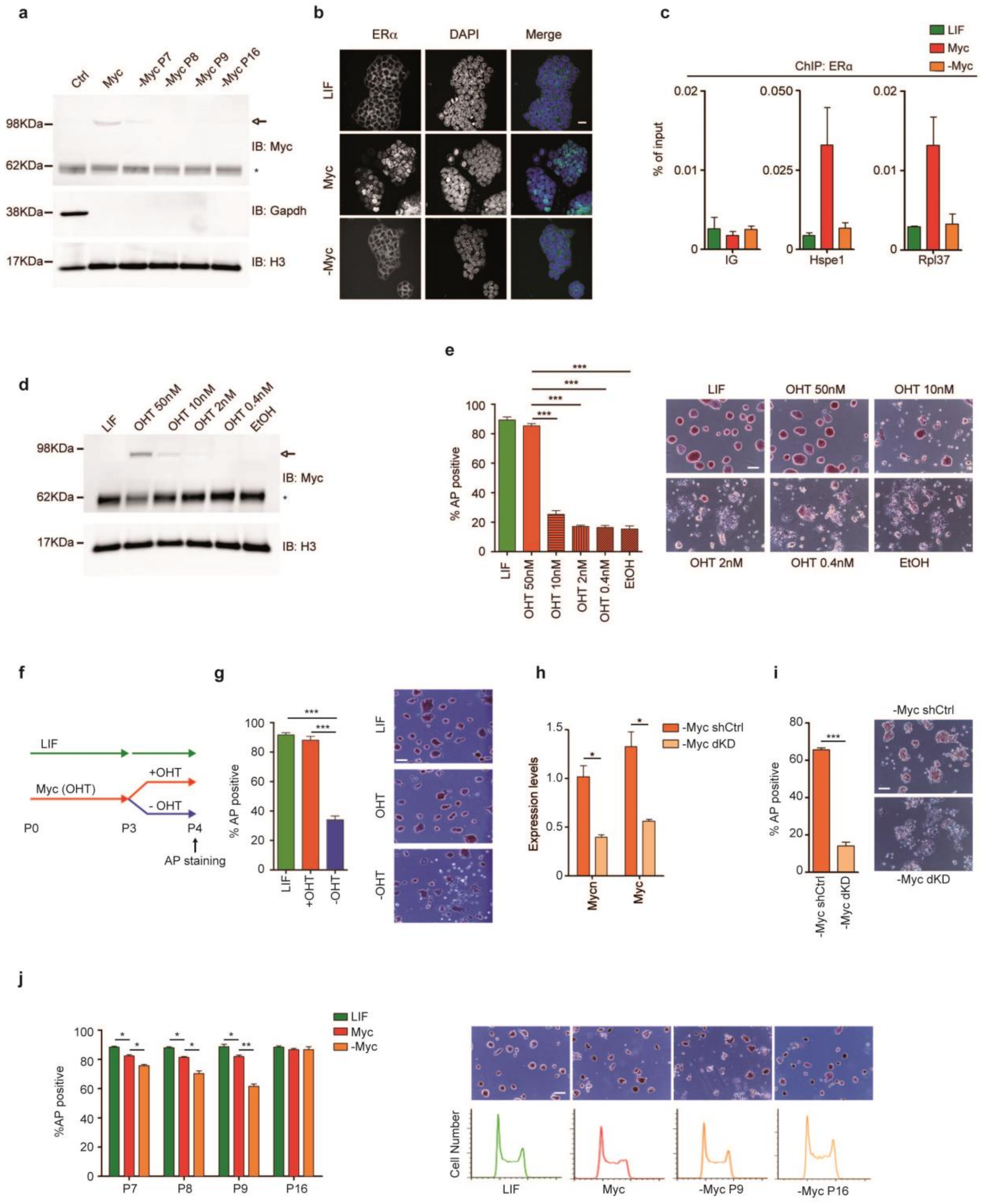

Fagnocchi_Supplementary Figure 9

Supplementary Figure 9. Myc-derived ESCs do not depend on the MycER fusion protein

(**a**) Expression of MycER in total protein extracts form LIF-ESCs (Ctrl) and nuclear protein fractions from Myc- and –Myc-ESCs at indicated passages. Immunostaining (IB) was performed using the indicated antibodies. The arrow indicates the MycER, while the asterisk indicates endogenous Myc protein. Gapdh and H3 were used as cytosolic and nuclear loading control, respectively. (**b**) Immunofluorescence analysis of MycER (ERα) in LIF-, Myc- and -Myc-ESCs. Scale bar, 50 µm. (**c**) ChIP of MycER (ERα) binding at known Myc targets, in LIF-, Myc- and -Myc-ESCs. IG = negative control. (**d**) Western blot analysis of nuclear protein fractions from LIF-ESCs or ESCs grown solely in presence of indicated amount of OHT. Immunostaining (IB) analysis was performed with indicated antibodies; the arrow indicates the MycER fusion protein, while the asterisk indicates endogenous Myc protein. H3 was used as loading control. (**e**) Alkaline Phosphatase (AP) staining of LIF-ESCs or ESCs grown solely in presence of indicated amount of OHT; scale bar 200 µm. (**f**) Schematic representation of the experiment. MycER ESCs were grown either in LIF- (green line) or Myc-dependency (Myc, red line). After three passages Myc-ESCs were either kept in the same medium (+OHT) or assessed for their ability to self-renew in an OHT-free culture medium for an additional passage (-OHT, blue line). (**g**) AP staining of LIF-dependent (LIF), OHT-dependent (+OHT) and OHT-depleted (-OHT) cells, obtained after only 3 passages in OHT-dependency; scale bar 200 µm. (**h**) Relative transcriptional level of Myc and Mycn assessed by qRT-PCR, in Myc-derived ESCs expressing either a control (shCtrl) or a double Myc and Mycn (dKD) shRNAs. (**i**) AP staining of Myc-derived ES cells expressing either a control (shCtrl) or a double Myc and Mycn (dKD) shRNAs and grown in absence of both LIF and OHT for three days; scale bar 200 µm. (**j**) AP staining of LIF-, Myc- and –Myc-ESCs generated in the MycER A2 clone. Representative images and the relative cell cycle profiles are shown. Scale bar, 200 µm. Data in (**c**), (**e**) and (**g-**

j) are means +/- s.e.m. (n=3). (*P<0.05, **P<0.01, ***P<0.001; Student's t test). Related to Figure 6.

a

-Myc P9 up-regulated genes (vs LIF)

IPA pathways

Term	P-value
Integrin Signaling	6.3E-12
PCP pathway	2.6E-09
ILK Signaling	3.9E-07
Wnt/β-catenin Signaling	1.7E-06
Regulation of the EMT Pathway	2.9E-06
Human Embryonic Stem Cell Pluripotency	1.8E-05

b

-Myc P9 down-regulated genes (vs LIF)

IPA pathways

Term	P-value
Role of JAK1, JAK2 and TYK2 in Interferon Signaling	8.4E-05
JAK/Stat Signaling	5.0E-04
Role of Nanog in Mammalian ESCs Pluripotency	2.0E-03
Mouse ESCs Pluripotency	1.0E-02
Transcriptional Regulatory Network in ESCs	1.0E-02

-Myc P16 up-regulated genes (vs LIF)

IPA pathways

Term	P-value
Regulation of the EMT Pathway	1.1E-06
FGF Signaling	2.0E-04
Wnt/β-catenin Signaling	5.9E-04
Integrin Signaling	1.5E-03
Mouse ESCs Pluripotency	1.8E-03
EGF Signaling	6.1E-03

-Myc P16 down-regulated genes (vs LIF)

IPA pathways

Term	P-value
Role of JAK1, JAK2 and TYK2 in Interferon Signaling	4.3E-02

c

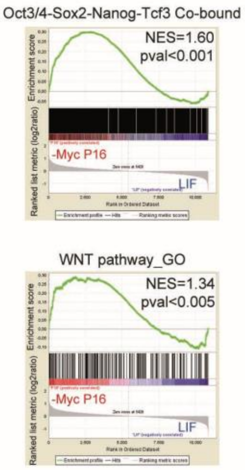

d

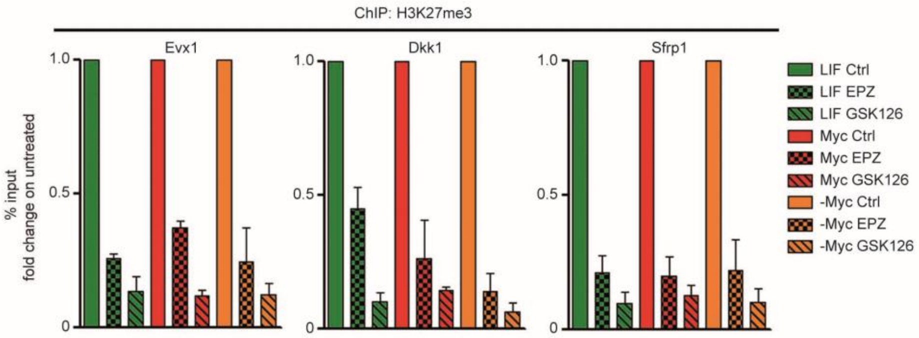

e

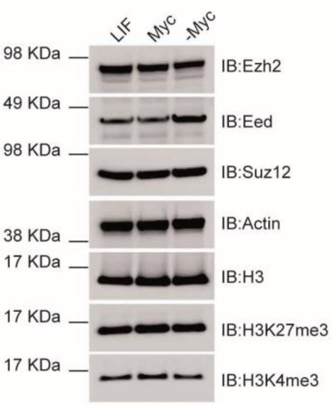

f

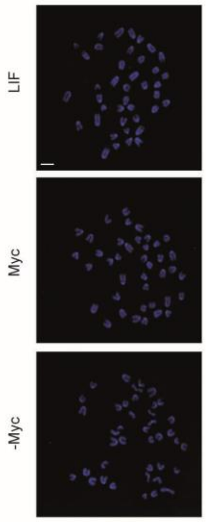

Fagnocchi_Supplementary Figure 10

Supplementary Figure 10. Characterization of Myc-derived ESCs

(**a-b**) Ingenuity Pathway Analysis (IPA) of differentially up-regulated (**a**) and down-regulated (**b**) genes between LIF- and Myc-derived ESCs at passage 9 (upper panels) or passage 16 (lower panels) (n=3). (**c**) Gene set enrichment analysis (GSEA) of Oct4/Sox2/Nanog/Tcf3 common targets (left) and Wnt pathway genes (right) in Myc-derived at passage 16 versus LIF-maintained ESCs (n=3). NES = normalized enrichment score. (**d**) ChIP analysis of the H3K27me3 deposition in presence of chemical inhibitors of PRC2 (EPZ and GSK126). Chromatin extracts were obtained from either the MycER ESCs maintained in LIF- or OHT-dependency (LIF and Myc, respectively) or the Myc-derived ESCs (-Myc). Chemical inhibitors of the PRC2 (EPZ and GSK126) were added to the culturing medium, as indicates. The level of H3K27me3 at the TSS of the indicated genes was measured by ChIP assay and shown as percentage of the input. Values relative to the untreated condition were set to 1 and all values were normalized accordingly. Evx1 was used a positive control. Data are means +/- s.e.m. (n=3). (**e**) Western Blot analysis of PRC2 proteins and histone H3 modifications in Myc-derived ESCs. Total protein extracts were collected from LIF-, Myc-maintained and Myc-derived ESCs and immunostaining (IB) analysis was performed using the indicated antibodies; β-actin and histone H3 were used as loading control. (**f**) Karyotype analyses of late passages MycER ESCs grown in presence of LIF, OHT (Myc) or in absence of both LIF and OHT after OHT withdrawal (-Myc), showing for each sample the normal number of chromosomes of mouse diploid nuclei (40 chromosomes). Karyotype analyses were performed on 15 individual nuclei of each cell type, resulting in 100% euploid cells. Chromosomes are fluorescently stained with DAPI; scale bar 10 μm. Related to Figure 6.

Figure 2b

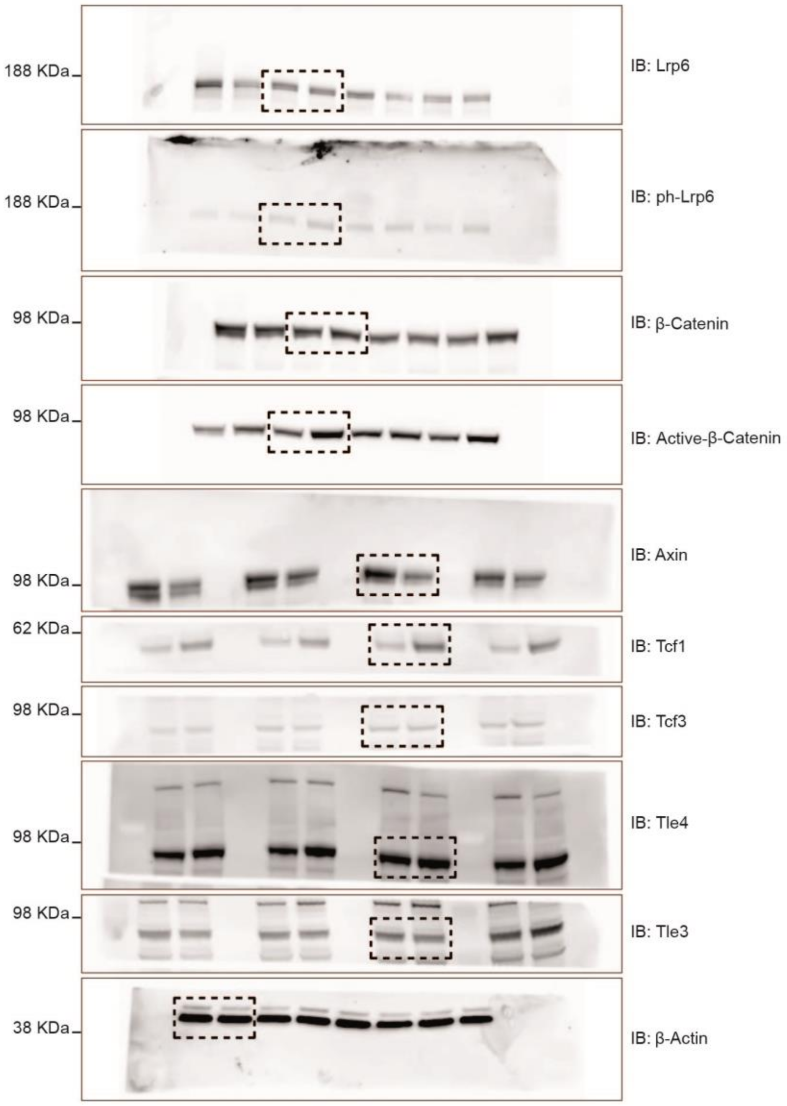

Figure 4b

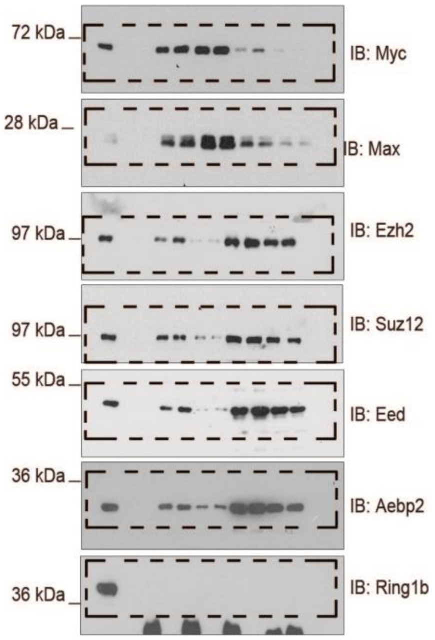

Figure 4c

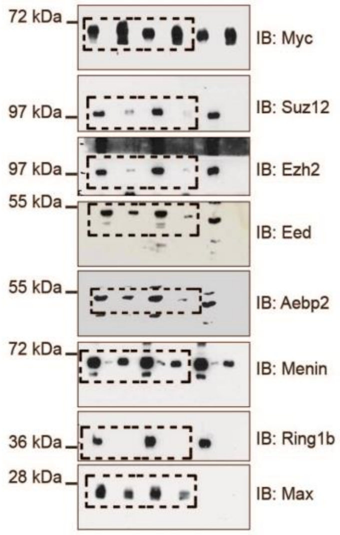

Figure 4d

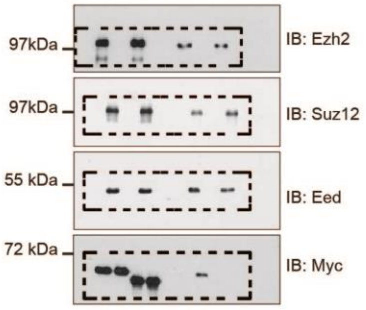

Figure 7c

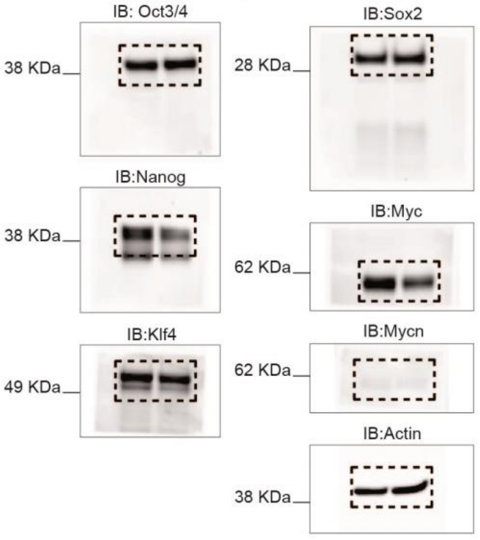

Fagnocchi_Supplementary Figure 11

Supp. Figure 1c

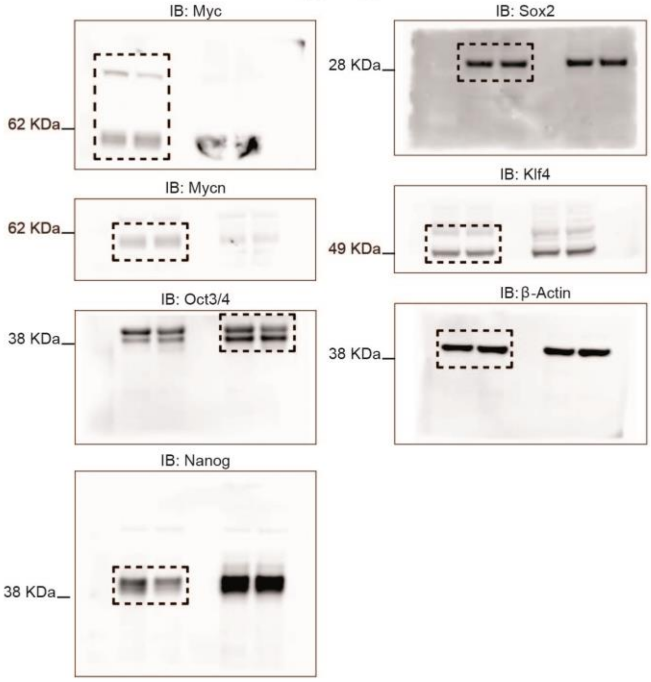

Supp. Figure 4a

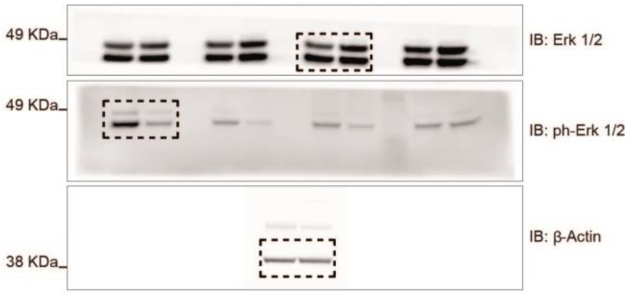

Supp. Figure 4g

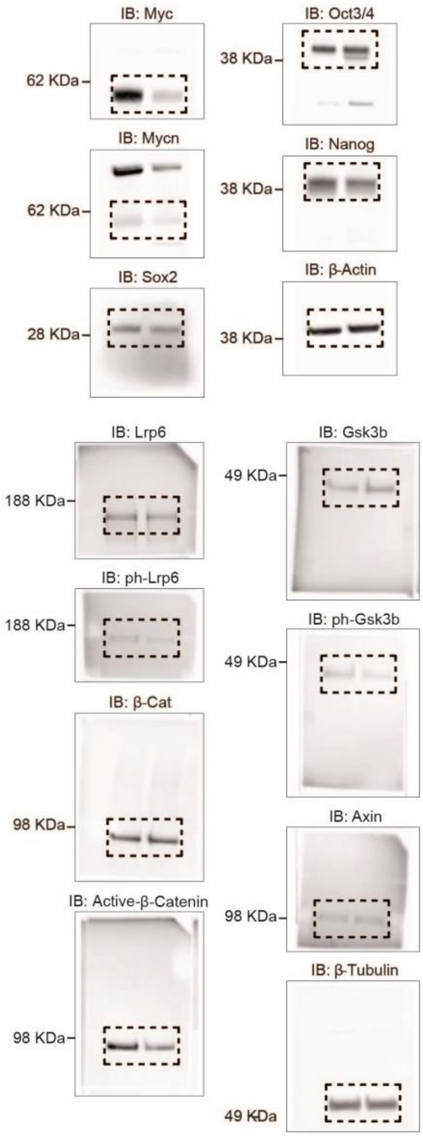

Supp. Figure 4c

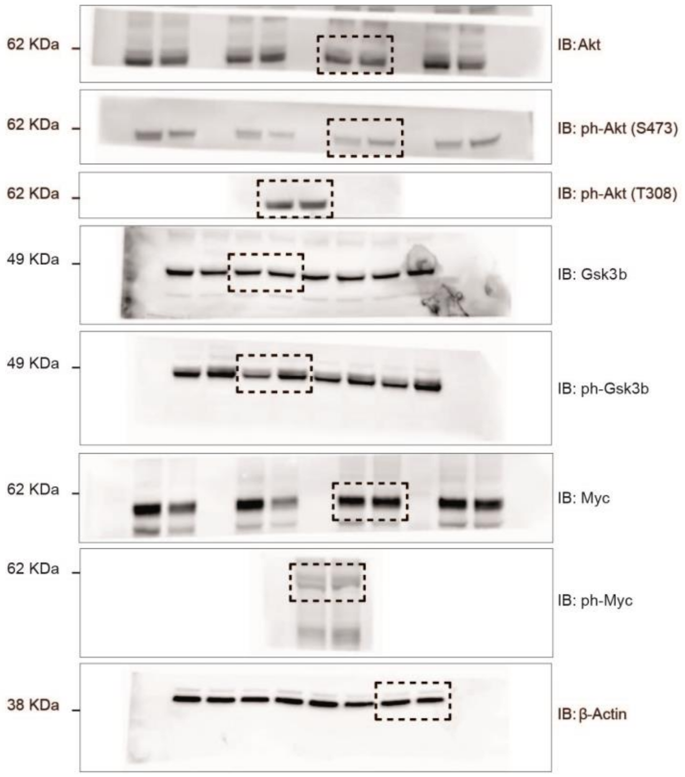

Fagnocchi_Supplementary Figure 11 (continued)

Supp. Figure 4h

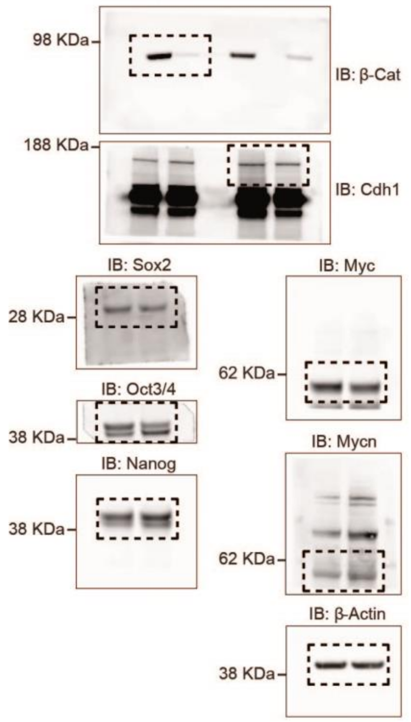

Supp. Figure 5f

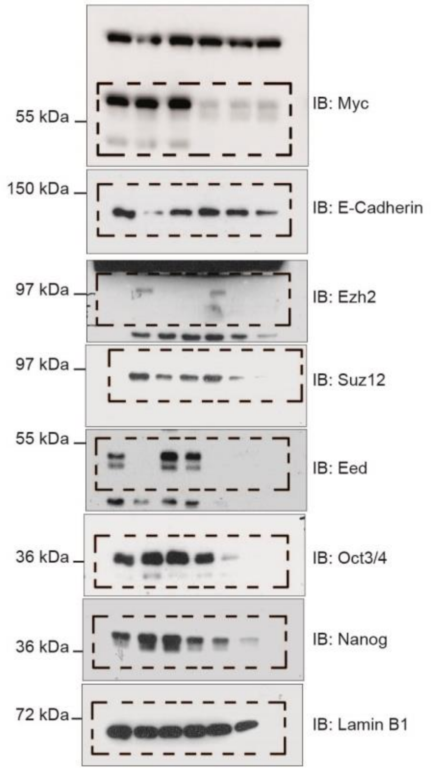

Supp. Figure 6e

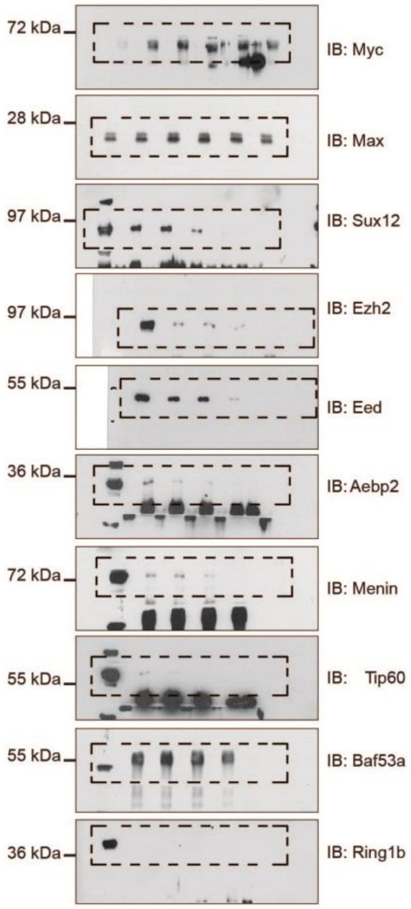

Supp. Figure 6f

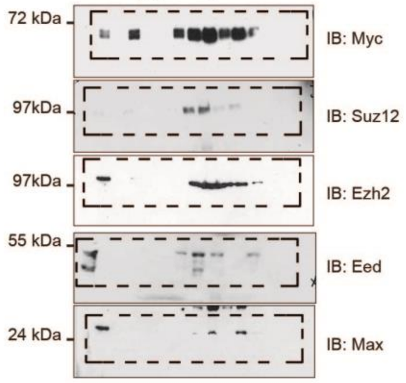

Supp. Figure 6h

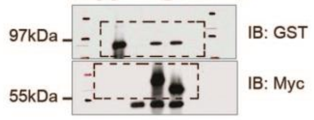

Supp. Figure 6i

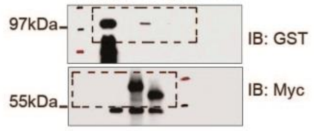

Fagnocchi_Supplementary Figure 11 (continued)

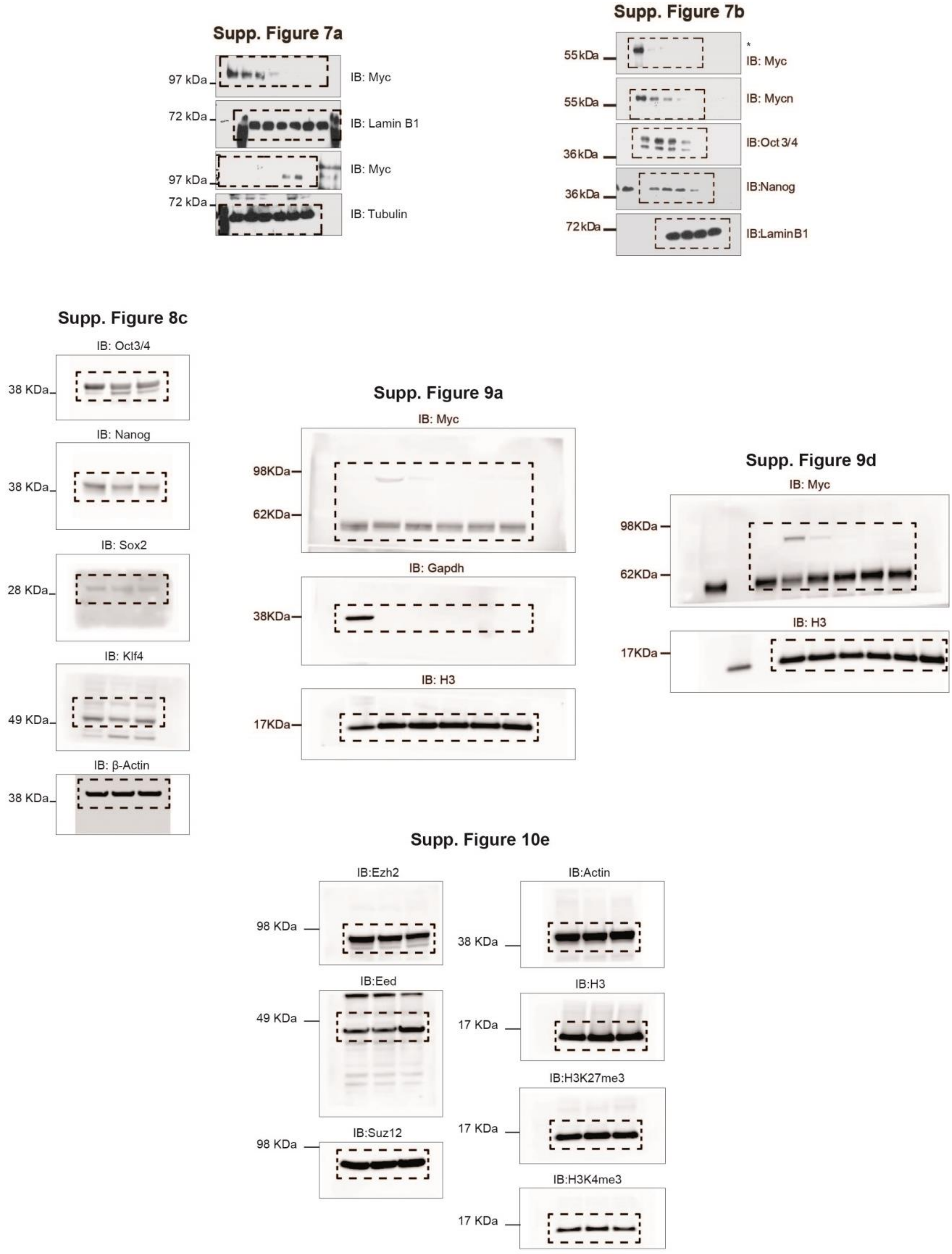

Fagnocchi_Supplementary Figure 11 (continued)

Supplementary Figure 11. Uncropped version of blots
